# Temporal progression of pathological features in an α-synuclein overexpression model of Parkinson’s disease

**DOI:** 10.1007/s00429-025-02959-9

**Published:** 2025-06-09

**Authors:** Andrea Vaquero-Rodríguez, Jone Razquin, Ane Murueta-Goyena, Cristina Miguelez, José Ángel Ruíz-Ortega, José Vicente Lafuente, Harkaitz Bengoetxea, Naiara Ortuzar

**Affiliations:** 1 Department of Neurosciences, Faculty of Medicine and Nursing, University of the Basque Country (UPV/EHU), Leioa, 48940 Spain; 2 Department of Pharmacology, Faculty of Medicine and Nursing, University of the Basque Country (UPV/EHU), Leioa, 48940 Spain; 3 Neurodegenerative Diseases Group, Biobizkaia Health Research Institute, Barakaldo, 48903 Spain

**Keywords:** Parkinson's disease, α-synuclein, Nigrostriatal pathway, Dopaminergic neurons, Axonal swellings, Microglia

## Abstract

Parkinson’s disease (PD) is a common neurodegenerative disorder, affecting 1–5% of individuals over 60, with a higher incidence in men. It is clinically characterized by progressive motor impairments, including rigidity, bradykinesia, tremors, and gait disturbances. The neuropathological hallmark of PD is the aggregation of α-synuclein (α-syn) into Lewy bodies (LB) and neurites (LN). Although α-syn plays essential physiological roles, its misfolding and accumulation drive neurodegeneration. In this study, we investigated the temporal progression and anatomical distribution of α-syn pathology using a bilateral adeno-associated virus serotype-9 (AAV9)-mediated α-syn overexpression model in rats. Disease-related features were analyzed at one, two and four months post-injection. Neuronal α-syn overexpression was confirmed as it co-localized predominantly with tyrosine hydroxylase (TH)-positive neurons, distinctly separate from glial markers. Behavioral assessment, immunofluorescence assays, stereological quantification, and optical densitometry revealed progressive motor impairments, dopaminergic neuronal loss in the substantia nigra pars compacta (SNpc), and decreased TH + fibers in the striatum and dendrites of the substantia nigra pars reticulata (SNpr). These changes were accompanied by increased microglial activation. Furthermore, axonal swellings in the striatum increased progressively over time, correlating with reductions in striatal TH optical density. By characterizing the temporal dynamics of α-syn-induced pathology, this study underscores the model’s relevance for PD research and highlights critical time windows for evaluating therapeutic interventions.

**Supplementary Information:**

The online version contains supplementary material available at 10.1007/s00429-025-02959-9.

## Introduction

Parkinson’s disease (PD) is the second most common neurodegenerative disease, affecting approximately 1–5% of individuals over the age of 60 (Pringsheim et al. 2014). Its incidence is 1.5 times higher in men compared to women (Wooten et al. 2004; Taylor et al. 2007; Armstrong and Okun 2020), and it is considered a multisystem neurodegenerative disorder (Braak et al. 2003).

Main symptoms of PD include rigidity, bradykinesia, tremors and gait disturbances. These clinical manifestations are attributed to the progressive loss of dopaminergic (DA) neurons in the substantia nigra pars compacta (SNpc), resulting in diminished dopamine levels within the basal ganglia (Forno 1996; Kaur et al. 2019). The disruption of the nigrostriatal pathway (Braak et al. 2003) is closely linked to the accumulation of intraneuronal protein aggregates, known as Lewy bodies (LBs) and Lewy neurites (LNs).

The most relevant neuropathological hallmarks of PD are these intracellular aggregates, which are composed of α-synuclein (α-syn) (Cerri et al. 2019; Hijaz and Volpicelli-Daley 2020). These inclusions are not restricted to the soma of DA neurons, as PD patients also exhibit dystrophic axons displaying a characteristic “string-of-beads” morphology (Kordower et al. 2013; Quintino et al. 2022). The formation of these axonal swelling is thought to result from the progressive aggregation of oligomeric α-syn fibrils, along with other protein components found in LB and LN (Kordower et al. 2013; Quintino et al. 2022).

Numerous studies have also demonstrated that α-syn overexpression and aggregation are associated with microglial activation in PD. This reactive state is associated with elevated levels of proinflammatory cytokines (Sanchez-Guajardo et al. 2010; Tremblay et al. 2015) and impaired microglial phagocytosis (Nayak et al. 2014; Choi et al. 2015). Post-mortem tissue analyses have further revealed microglial activation not only in the substantia nigra (SN) but also in other brain regions, including the putamen, hippocampus, and cerebral cortex (Imamura et al. 2003). These findings support the notion that glial dysfunction may play a central role in initiating and sustaining neuroinflammatory responses across the brain, thereby contributing to the neuronal damage characteristic of PD.

Given its central role in PD, α-syn aggregation is widely regarded as a promising therapeutic target (Dehay and Fernagut 2016). Its involvement in disease progression has led to the development of several animal models aimed at elucidating how α-syn aggregation contributes to synaptic dysfunction and DA neuron degeneration (Dehay et al. 2015). In transgenic α-syn models, both motor impairments (Chesselet and Richter 2011) and disruptions in dopamine homeostasis have been documented, affecting its release, reuptake and overall tissue content (Kurz et al. 2010; Lam et al. 2011). Alternative modeling approaches include viral vector-mediated overexpression of α-syn or the local injection of pathogenic pre-formed α-syn fibrils (PFFs). Compared to the adeno-associated virus (AAV)-α-syn model, the PFFs model induces a gradual Lewy body-like pathology that affects not only nigral DA neurons but also more widespread brain regions (Gómez-Benito et al. 2020; Björklund et al. 2022). However, in this model, substantial neuronal loss emerges only after five to six months, with degeneration in rats displaying considerable variability and often insufficient to produce clear motor deficits (Patterson et al. 2019; Björklund et al. 2022). In contrast, the AAV-α-syn model results in more rapid and pronounced neurodegeneration than both transgenic and PFF-based models, although it requires high α-syn expression levels to induce marked neuronal loss (Gómez-Benito et al. 2020; Björklund et al. 2022). Moreover, AAV vectors are generally preferred over lentiviral (LV) vectors due to their enhanced efficiency in producing rapid and widespread neurodegeneration (Lauwers et al. 2003, 2007; Bourdenx et al. 2015; van der Perren et al. 2015).

While AAV-mediated unilateral α-synuclein overexpression models have been extensively employed to study PD, the temporal dynamics of neuropathological progression remain poorly defined in bilateral models. This study aims to systematically delineate the time course of pathological alterations and to determine the optimal window for assessing neuroprotective strategies, as bilateral models more faithfully replicate the progressive pathology observed in PD patients.

## Methods

### Animals and experimental groups

A total number of 108 male Sprague Dawley rats (8 weeks old, weighing 240–260 g), were used in this study. Animals were housed under controlled conditions with a 12 h light/dark cycle and had ad libitum access to both food and water.

The rats were initially divided into three experimental groups based on stereotactic injection. 36 rats were assigned to the α-syn group and received bilateral injections of the recombinant viral vector rAAV9-CMVie/SynP-WPRE into the SNpc. Another 36 rats received a bilateral injection with the empty viral vector rAAV9-CMVie/SynP-wtsyn-WPRE into the SNpc and were allocated to the sham group. The remaining 36 rats were assigned to the control group, consisting of healthy animals that did not receive any injection.

After the surgical procedure, animals within each experimental group were further divided into three subgroups according to postoperative survival time. In each experimental group, 12 animals were sacrificed at 1 month, another 12 at 2 months, and the remaining 12 at 4 months post-injection.

All experimental procedures were conducted in accordance with the European Communities Council Directive on “The Protection of Animal Uses for Scientific Purposes” (210/63/EU) and Spanish Law (RD 53/2013) for the care and use of laboratory animals. The study was approved by the Bioethical Committee for Animal Research of the University of the Basque Country (Spain) (UPV/EHU, CEEA, ref M20/2020/241; CEIAB, ref M30/2021/242).

### Stereotactic injection

Animals underwent bilateral stereotactic injection to induce α-syn overexpression. Animals were anesthetized with isoflurane (4% for induction and 1,5 − 2% for maintenance) and then, were placed in the stereotactic head frame (David Kopf^®^ Instruments, Tujunga, California, USA, model 957). The stereotactic coordinates for the SNpc were AP= -5.3 mm, ML= -2.0 mm, DV= -7.2, relative to bregma.

All animals received an injection of 1 µL of viral vector (rAAV9-CMVie/SynP-wtsyn-WPRE or rAAV9-CMVie/SynP-WPRE) at a viral title of 1.07 × 10^13^ gcp/ml. The scalp was incised, and the skull was exposed. A small hole was drilled, and injections were performed using a glass capillary to minimize tissue damage, at a rate of 0.125 µL per minute. After the infusion was completed, the needle was left in place for an additional 5 min to allow diffusion of the viral vector in the injection site before being slowly retracted. For post-surgical recovery, meloxicam (2 mg/kg) and saline (0.9%, 10 mL/kg) were administered subcutaneously.

### Open field test

Spontaneous locomotor activity was assessed one, two and four months after injury using an open field (OF) arena (Panlab, Spain). Each rat was placed in the centre of the arena, and after a two-minute adaptation period, its activity was recorded for 10 min. The detection of global activity, locomotion and mean velocity was fully automated using the custom-designed software Actitrack (Panlab, Spain).

### Tissue sample collection

Six rats per experimental group were anesthetized with pentobarbital (60 mg/kg, i.p., Nembutal, Ceva Santé, Belgium) and transcardially perfused with 4% paraformaldehyde in phosphate-buffered saline (PBS 0.1 M, pH = 7.4). Brains were subsequently extracted and post-fixed overnight in the same solution.

After fixation, brains were cryopreserved in 30% sucrose diluted in 0.1 M PBS for at least 48 h. Coronal  sections(40 μm thick) were obtained with a freezing microtome (HM 430, Microm^®^), and stored at -20 ºC in a cryoprotective solution containing 30% ethylene glycol and 26% glycerol in PBS until further processing.

### Immunofluorescence assays

Double immunofluorescence was performed on free-floating  sections (2–6 animals per group; 8 slices per animal) using an antibody against α-syn (mouse monoclonal Ab27766; 1:20000; Chemicon, USA; RRID: AB_727020) in combination with either tyrosine hydroxylase (TH) (rabbit polyclonal Ab152; 1:1000; Chemicon, USA; RRID: AB_390204), glial fibrillary acidic protein (GFAP) (mouse monoclonal G-3893; 1:1000; Sigma-Aldrich, Germany; RRID: AB_477010), or Ionized Calcium-Binding Adapter Molecule 1 (Iba-1) (rabbit polyclonal Ab178846; 1:1000; Abcam, UK; RRID: AB_2636859). Sections were washed twice for 5 min in 0.1 M PBS, followed by blocking in PBS containing 5% Normal Horse Serum (NHS) for 2 h at room temperature.

Subsequently, sections were incubated overnight at room temperature with the primary antibodies, diluted in the same blocking solution, under continuous stirring. After incubation, sections were washed in 0.1 M PBS and incubated for 1 h with Alexa Fluor 594 (A11005; 1:400; Invitrogen, Massachusetts, USA; RRID: AB_141372) and Alexa Fluor 488 (A11008; 1:400; Invitrogen, Massachusetts, USA; RRID: AB_143165) secondary antibodies diluted in 0.1 M PBS containing 0.05% Triton X-100. Finally, sections were washed three times in 0.1 M PBS and mounted on gelatin-coated slides for imaging with a confocal microscope (Zeiss LSM800).

The spatial distribution of α-syn was further assessed by immunofluorescence in 14 free-floating serial coronal sections, using the same α-syn antibody (mouse monoclonal Ab27766; 1:20000; Chemicon, USA; RRID: AB_727020). The distribution pattern was analyzed from the injection site across multiple brain regions, including the superior colliculus and the periaqueductal gray in the midbrain, the medial geniculate nucleus of the thalamus, the retrosplenial cortex, and the prefrontal cortex.

### Co-localization of TH/α-syn in SNpc: efficacy and specificity

Co-localization of TH and α-syn in SNpc neurons was evaluated in four α-syn animals one month post-injection. For each animal, four coronal brain sections were analyzed, with each SNpc evaluated independently.

Image acquisition was conducted using a Nikon Ti-U conventional fluorescence microscope equipped with a Nikon DS-Qi2 digital camera (Nikon, Japan) at the SGIker core facilities of the University of the Basque Country (UPV/EHU). Z-stacks were acquired using a 20× objective (CFI Plan Apo Lambda 20×/0.75, WD 1.0 mm) with a step size of 2.5 μm. Image resolution and contrast were enhanced through Richardson–Lucy deconvolution.

Maximum intensity projection (MIP) images were generated using FIJI (NIH). Images were converted to 8-bit grayscale, and binary masks for TH- and α-syn-positive neurons were generated by applying intensity thresholding and removing outliers (Pixels = 15, Threshold = 50). Adjacent structures were separated using the watershed algorithm. Automated quantification of immunopositive neurons was performed using FIJI´s “Analyze Particles” (size = 30-infinity).

To quantify co-localization, binary masks for TH and α-syn were merged, and double-labeled neurons were manually counted using the “Cell Counter” tool in FIJI. Co-localization was quantified as Efficacy, defined as the proportion of TH + neurons that also expressed α-syn (N (α-syn /TH)/ N (TH)), and Specificity, defined as the proportion of α-syn + neurons that were also TH+ (N(α-syn /TH)/N(α-syn)).

### Immunohistochemistry

Immunohistochemistry was carried out on free-floating sections using antibodies against TH (rabbit polyclonal AB152; 1:1000; Chemicon, USA; RRID: AB_390204) and Iba-1 (rabbit polyclonal AB178846; 1:1000; Abcam, UK; RRID: AB_2636859). Sections were pretreated with 1:30 H₂O₂ in 0.1 M PBS for 20 min, followed by washes in PBS. Then blocking was performed for 2 h in a solution containing 0.1 M PBS, 1:200 NHS, and 1:200 Triton X-100. Sections were then incubated overnight at room temperature with the primary antibody diluted in the same blocking solution, under continuous agitation. The following day, sections were washed in 0.1 M PBS and incubated for 1 h with a biotinylated secondary antibody (anti-rabbit Vecstain ABC Kit; 1:200, Vector Laboratories, Newark, USA; RRID: AB_2336810) in 0.1 M PBS containing 0.05% Triton X-100, under gentle shaking. Next, the immunoperoxidase ABC complex (Vecstain ABC Kit, 1:200, Vector Laboratories, Newark, USA) was applied in 0.1 M PBS with 0.05% Triton X-100 for 1 h under continuous stirring. Finally, after three washes in PBS, the immunoreaction was visualized using a 3,3′-diaminobenzidine (DAB) substrate solution composed of 10 µl DAB, 1 ml PBS, and 4 µl H₂O₂. Processed sections were then mounted on gelatin-coated slides for microscopic analysis and quantification.

### Unbiased stereology

Quantification of tyrosine hydroxylase-positive (TH+) cells in the SNpc and TH + dendrites of the substantia nigra pars reticulata (SNpr) was performed using unbiased stereology in 6 animals per group. The Mercator image analysis system (Explora-Nova, La Rochelle, France) was employed in combination with a digital camera connected to an Olympus BX51 microscope, equipped with a three-axis motorized stage.

The optical fractionator method was then used to estimate the total number of cells based on sampled cells within a set of virtual counting spaces, equally spaced in the X, Y, and Z directions, ensuring unbiased estimation. The total number of cells in the 40 μm thick-sections was calculated using the formula: N = ΣQ × (1/ssf) × (1/asf) × (1/hsf), where Q represents the actual number of counted cells, and N is the total estimated number of cells. The section sampling fraction (ssf) used in this study was 1/12.

Regions of interest were first delineated using a 4x objective, ensuring accurate identification of anatomical boundaries. Depending on the analyzed area (SNpc or SNpr), different grid and counting frame sizes were applied. In the SNpc, a counting frame of 50 × 50 μm with a grid spacing of 70 × 70 μm was used, incorporating a 5% guard zone. For estimating dopaminergic fiber length in the SNpr, the optical fractionator method was adapted by replacing optical dissectors with “spaceballs” (spherical dissectors), using a counting frame radius of 50 μm and a grid spacing of 450 × 450 μm. Quantification of immunopositive cells and fibers was performed using a 40x objective, following stereological counting guidelines. Both hemispheres of approximately 7–8 sections per animal were analyzed.

Finally, volume estimation was calculated using the Cavalieri method, and cell and fiber densities were determined by calculating the number of cells or fiber length, respectively, per cubic millimeter (mm³).

### Optical densitometry of tyrosine hydroxylase-positive fibers

Optical density (OD) analysis was performed to assess the extent of dopaminergic denervation induced by α-syn overexpression in the striatum. For that purpose, 7–8 coronal sections of the striatum per animal and 6 animals per group were digitized using an EPSON V700 scanner at a resolution of 6400 dpi. In each section, the dorsal striatum was delineated, and the mean OD was quantified for both hemispheres, using the cortex as a background reference. Image analysis was conducted using FIJI software (Schindelin et al. 2012).

### Quantification of axonal swelling and microglial activation

Quantification of axonal swellings and microglial activation was carried out using an optical microscope (Motic BA410E Series) equipped with a 20x objective and a digital camera.

To evaluate axonal swellings in the striatum of α-syn-overexpressing animals over time, a semi-quantitative analysis of TH + immunostaining was performed. For each animal, three images per hemisphere were acquired from 7 to 8 coronal striatal sections. Image processing was conducted using FIJI (NIH), where images were converted to 8-bit grayscale, background was smoothed, and binary segmentation was applied using the watershed algorithm to isolate individual structures. The area occupied by TH + axonal aggregates was measured relative to the total image area (mm²), and the mean value per animal was calculated.

To quantify microglial activation, Iba-1-positive area in the SN and striatum was analyzed using an automated image processing workflow. A custom macro developed in FIJI (ImageJ) was used to ensure consistency across samples. Four animals per group were analyzed, with four sections per animal and three images per section. Images were converted to 16-bit grayscale, background noise was reduced using a rolling ball subtraction algorithm (radius = 60 pixels), followed by a median filter (radius = 8 pixels) to eliminate high-frequency noise. Contrast was enhanced using a 0.20% saturation limit. Binary mask were generated via automatic thresholding using the Moments algorithm with the “Black Background” option enabled. For each image, total area and Iba-1-positive area were recorded, and the percentage of positive staining was calculated for group comparisons. All processed images were saved in TIFF format for visual inspection and validation of the analysis pipeline.

In both analyses, the primary outcome was the area occupied by immunopositive structures expressed as a proportion of the total image area.

### Statistical analysis

The Kolmogorov-Smirnov test was used to assess the normality of the data obtained, while Levene’s test was applied to evaluate homogeneity of variances. Significant differences between the experimental groups were determined using one-way analysis of variance (ANOVA) with Tukey’s post-hoc corrections for homogeneous variances, or Tamhane T2 for heterogeneous variances (SPSS Statistics 29.0, IBM, Spain). Pearson correlation coefficient was calculated to examine the statistical relationship between global activity and weight, as well as between sriatal optical density and axonal swellings across the three time points.

Temporal progression in both the behavioral and morphological data was analyzed using R Studio (version 01.07.2022). For the longitudinal analysis of behavioral data, linear mixed-effects models were applied to account for intra-animal correlation, using the lme4 and lmerTest packages. Fixed effects included time since surgery and experimental group, with a random intercept for each animal. To compare the progression of OF parameters between groups, an interaction term between time and group was included. Multivariate linear regression analyses were fitted to evaluate changes in TH and Iba-1 reactivity over time. Unlike the behavioural data, this model considered independent animal samples at each time point. Independent predictors included time, group, and time x group interaction term.

## Results

### Analysis of locomotor activity

**Fig. 1:**
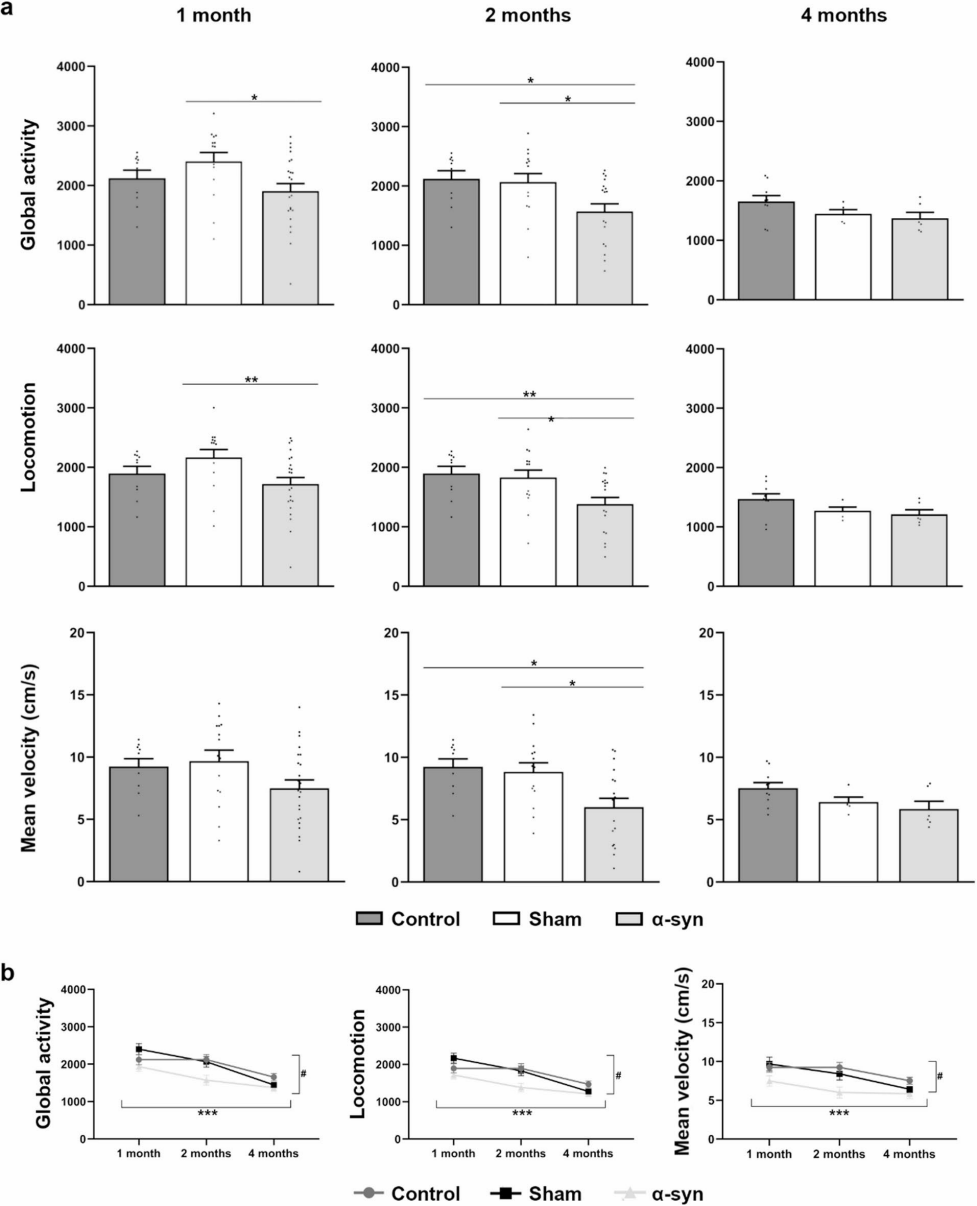
Open Field test in different experimental groups (control, sham, and α-syn) at one, two, and four months post-injection. Spontaneous global activity **(a)**, locomotion **(b)**, and mean velocity **(c)** were measured in an Open Field arena. Activity detection was fully automated using custom-designed software Actitrack (Panlab, Spain). Data are presented as mean ± SEM. *p < 0.05, **p < 0.01 (one-way ANOVA with post hoc corrections; *n* = 12 in each experimental group). **(d)** Temporal analysis assessing the progression of global activity. Data are presented as mean ± SEM. ***(time) *p* < 0.001; # (α-syn vs. control) *p* < 0.05 (multivariate linear regression analysis)

The α-syn overexpression group displayed marked motor deficits, evidenced by significant reductions in overall activity, locomotion, and velocity compared to both control and sham groups (Fig. 1a). These impairments were consistently observed at all analyzed time points, with statistically significant differences emerging at one and two months post-injection (Fig. 1a; **Online Resource 1** and **2**). By contrast, no statistical differences were detected at four months post-injection (Fig. 1a; **Online Resource 2**). These results support the progressive motor dysfunction induced by α-syn overexpression, reinforcing its impact on movement-related behaviors.

Temporal analysis revealed a significant decline in overall activity (*p* < 0.001), locomotor activity (*p* < 0.001), and mean velocity (*p* < 0.001) over time across all groups (Fig. 1b). In the α-syn group, motor performance progressively deteriorated, particularly in comparison to the control group (group × time interaction, *p* = 0.020). Although the sham group also exhibited a decline over time, its progression did not differ significantly from that of the α-syn group, suggesting a possible contribution of the surgical procedure to the observed effect. The control group remained stable until four months, when a decline was observed (Fig. 1b).

**Fig. 2:**
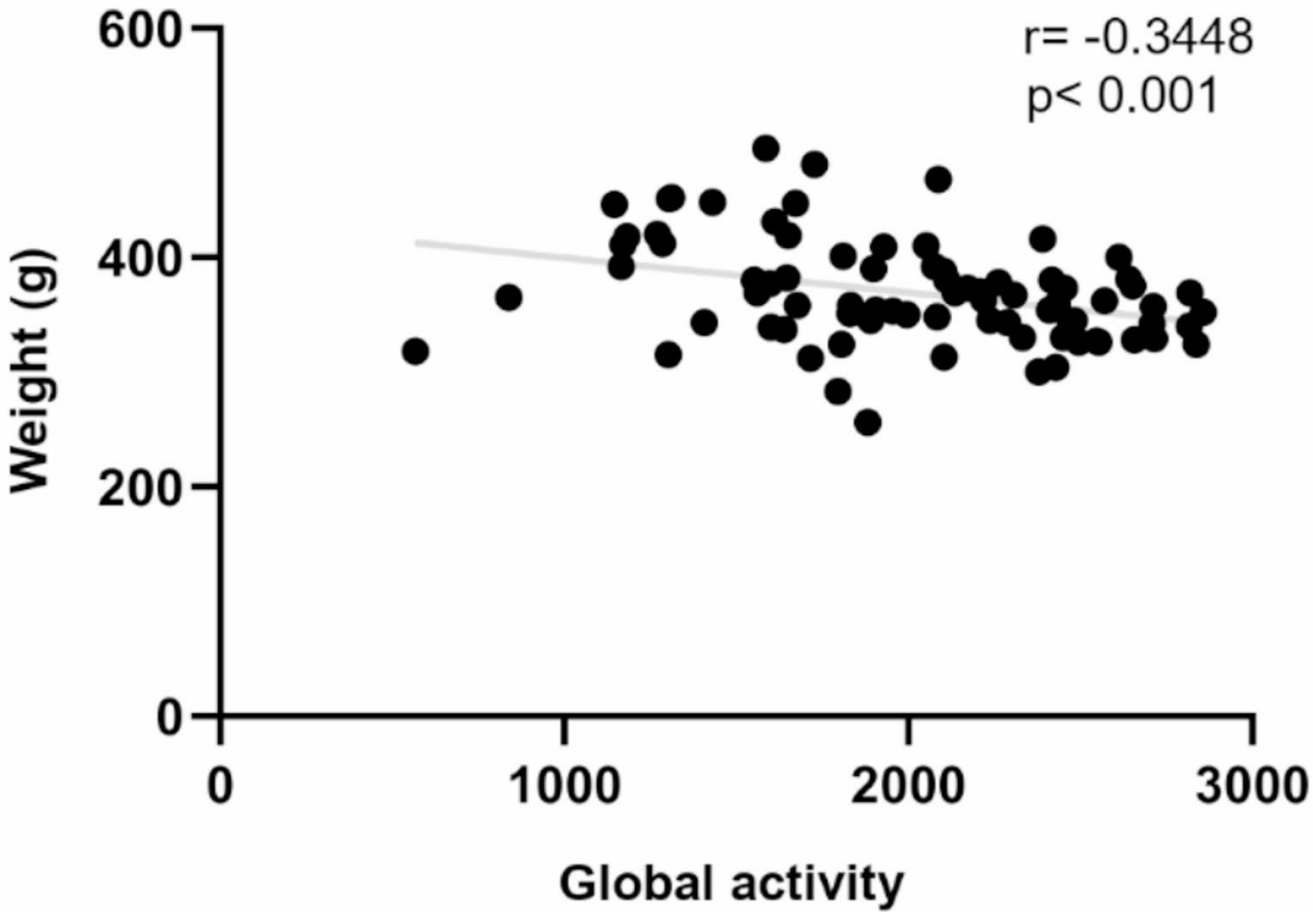
Correlation analysis to assess the relationship between the global activity measured in Open Field test and weight of the animals. Pearson correlation coefficient (*r* = -0.3448) and p-value (*p* < 0.001) shows a negative and significant correlation between overall activity and weight (*n* = 108)

Due to the absence of differences between experimental groups at four months and the general decline observed in all parameters at this time point, a correlation analysis was conducted to explore the potential relationship between the animals’ overall activity and body weight (Fig. 2). The results showed significant correlation (*r* = -0.3448; *p* < 0.001).

### α-Synuclein co-localization with dopaminergic neurons and glial markers in the nigrostriatal pathway

**Fig. 3:**
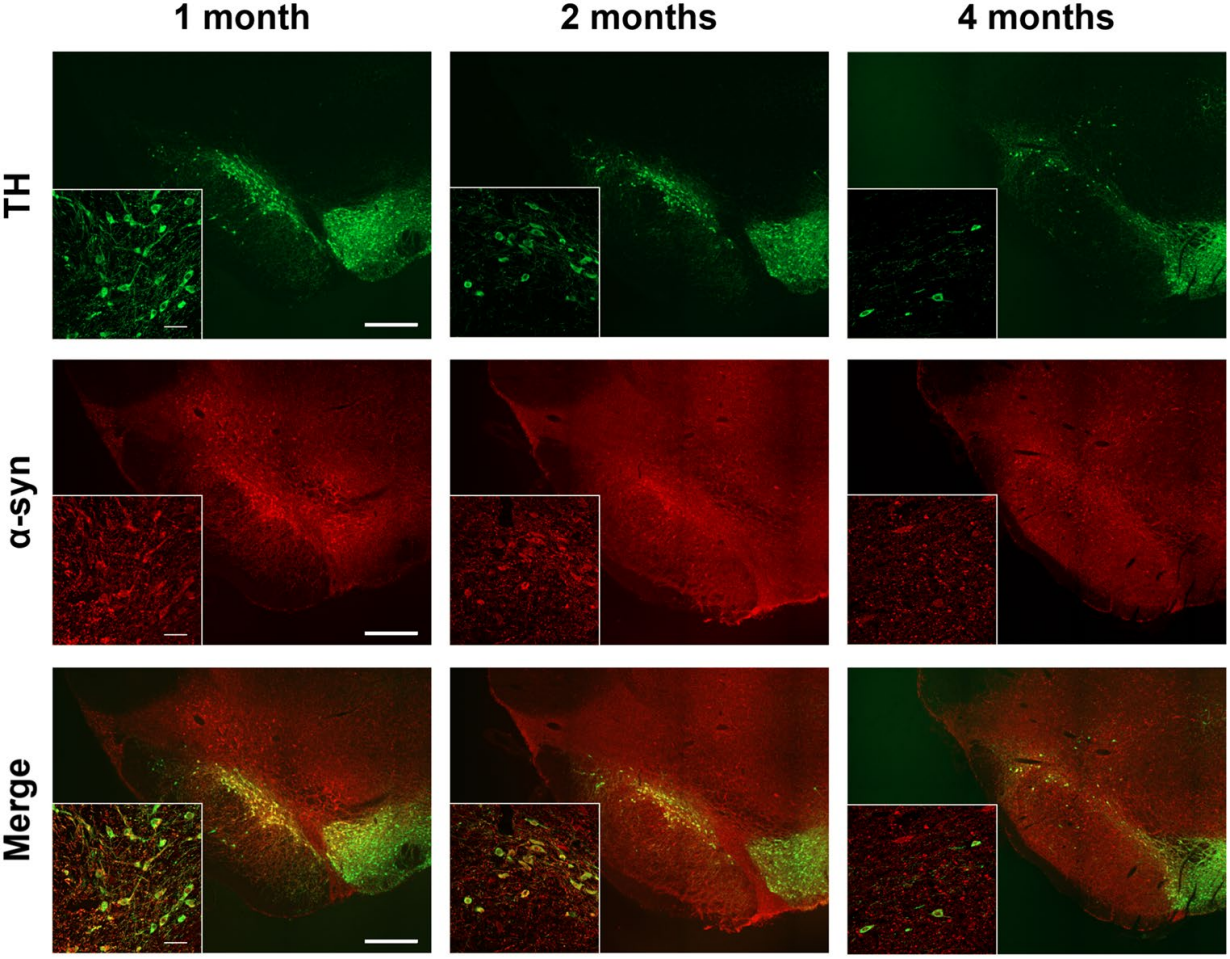
Representative double immunofluorescence images of the SN from α-syn animals showing positive labelling for TH (green), α-syn (red), and their co-expression (yellow) at one, two, and four-months post-injection (*n* = 6 in each analyzed time point). Scale bar: 500 μm and 50 μm

**Fig. 4:**
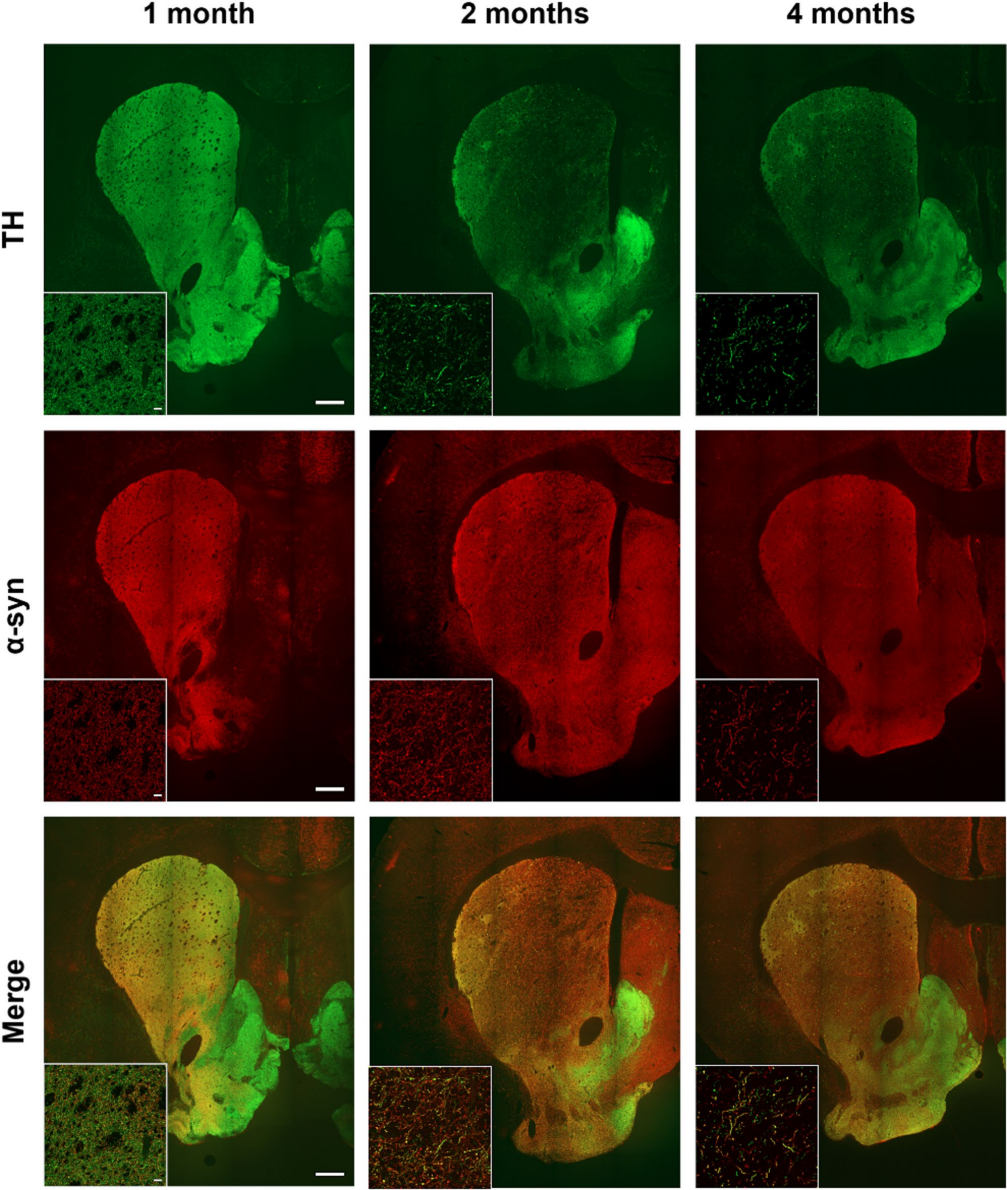
Representative double immunofluorescence images of the striatum from the α-syn animals showing positive labelling for TH (green), α-syn (red), and their co-expression (yellow) at one, two, and four months post-injection (*n* = 6 in each analyzed time point). Scale bar: 500 μm and 50 μm

The spatial correlation between α-syn expression and TH + dopaminergic neurons was assessed by double immunofluorescence. Co-localization of α-syn with TH + neurons was observed in both the SNpc (Fig. 3) and striatum (Fig. 4). Notably, α-syn overexpression was restricted to the α-syn group (Figs. 3 and 4), as no immunoreactivity for human α-syn was detected in either control (**Online Resource 3** and **4**) or sham (**Online Resource 5** and **6**) groups. A time-dependent decrease in the TH/α-syn co-expressing neurons in the SNpc and in the α-syn-positive fibers in the striatum was also observed, suggesting progressive dopaminergic neuronal loss and axonal degeneration induced by α-syn overexpression (Figs. 3 and 4).

**Fig. 5:**
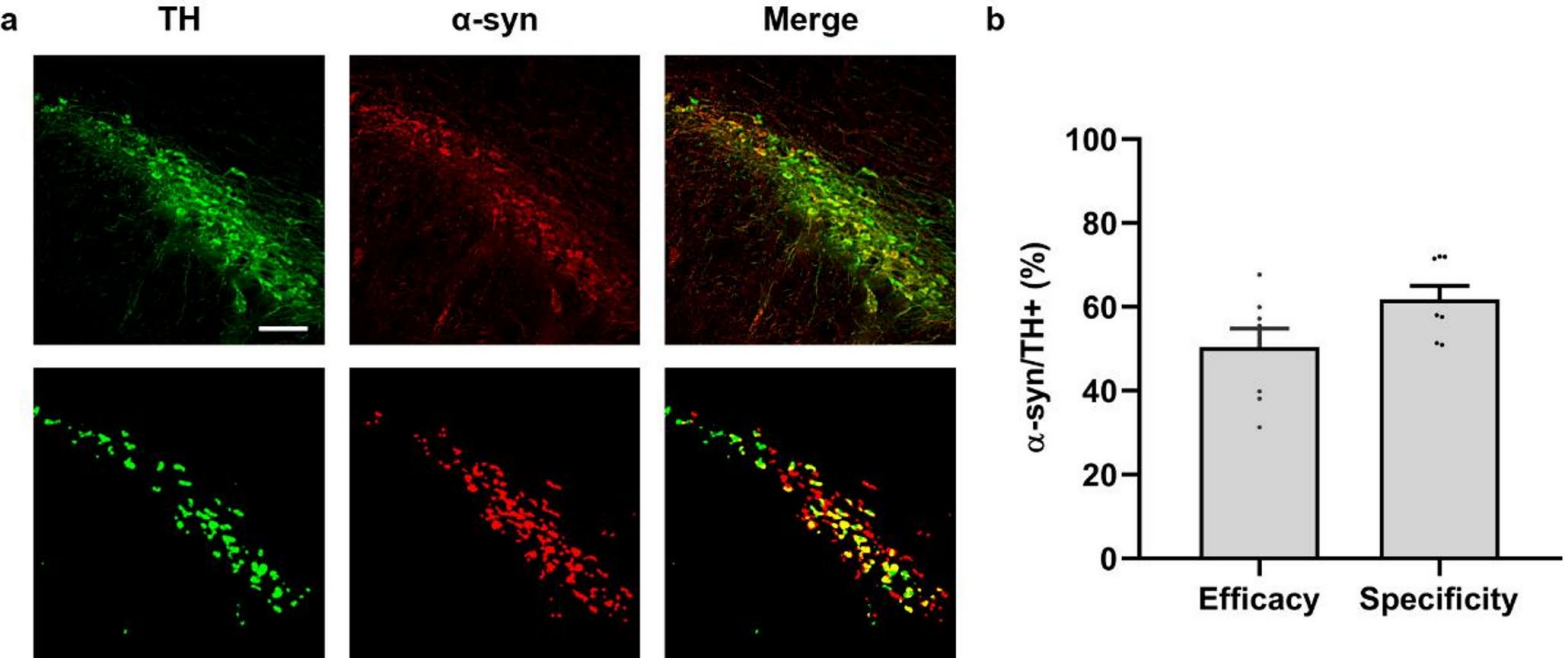
Co-localization analysis of TH and α-syn-positive neurons in the SNpc at one month post-injection (*n* = 4). **(a)** Representative double immunofluorescence images showing TH (green), α-syn (red), and their co-localization (yellow), along with corresponding binary masks (lower pannel). Scale bar: 200 μm. **(b)** Quantification of colocalization expressed as efficacy (N(α-syn⁺/TH⁺) / N(TH⁺)) and specificity (N(α-syn⁺/TH⁺) / N(α-syn⁺))

To further evaluate the viral vector performance, the efficacy and specificity of AAV-mediated transduction of TH + neurons were quantified one month post-injection. The viral vector achieved a transduction efficacy of 50% and a specificity of 62% for TH + neurons (Fig. 5).

**Fig. 6:**
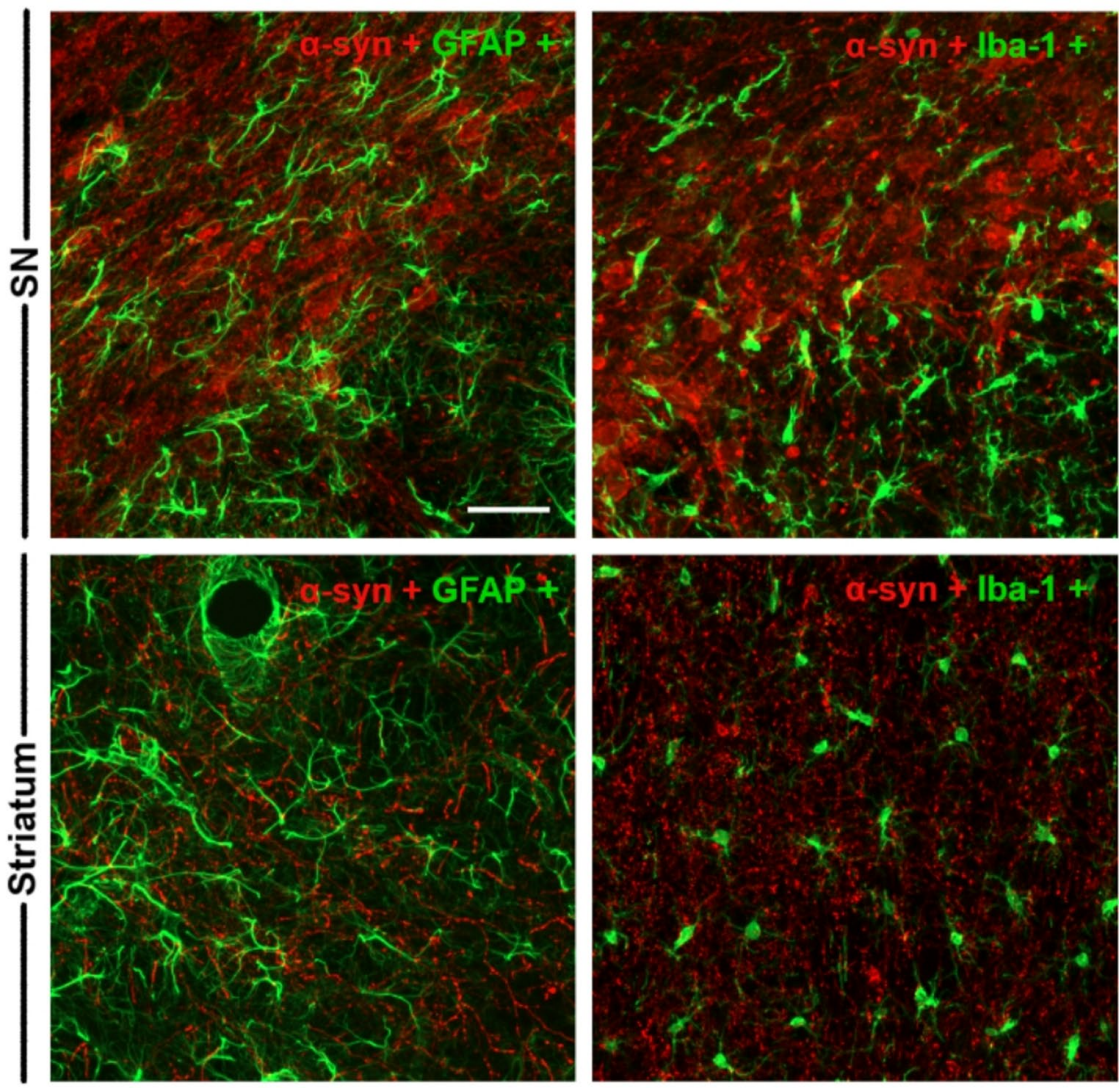
Representative double immunofluorescence images of the SN and striatum of α-syn animals. Positive labeling for GFAP and Iba-1 is shown in green, and α-syn in red. The images show no co-expression of α-syn with astrocytes and microglia (*n* = 2 in each analyzed time point). Scale bar: 50 μm

In addition, to determine whether α-syn expression extended to non-neuronal cell types, double immunofluorescence for astrocytes (GFAP) and microglia (Iba-1) was performed. No co-localization of α-syn with either cell type was detected in the SNpc or the striatum (Fig. 6), indicating that the AAV employed in our model predominantly targets TH + neurons, though it may also transduce other neuronal populations within the SNpc.

### α-Synuclein overexpression and diffusion in the brain

**Fig. 7:**
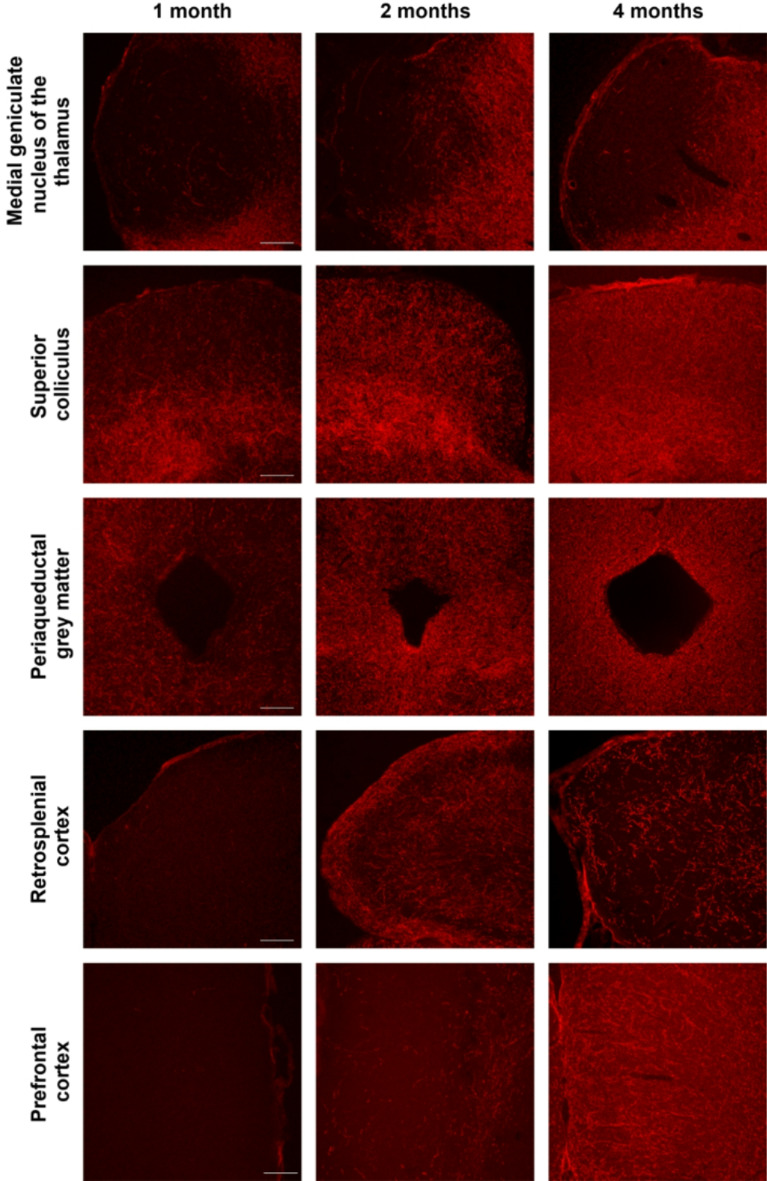
Representative immunofluorescence images of α-syn (red) in serial coronal brain sections corresponding to the medial geniculate nucleus of the thalamus, the superior colliculus, the mesencephalic aqueduct, the retrosplenial cortex, and the prefrontal cortex. The images show α-syn positivity in most areas one, two, and four months after injection, with the exception of the prefrontal cortex, where positivity is observed from 2 months post-injection (*n* = 2 in each analyzed time point). Scale bar: 200 μm

Following confirmation of α-syn co-localization with TH + neurons of the nigrostriatal pathway, the spatial distribution of α-syn was further examined (Fig. 7). Serial coronal brain sections were analyzed to trace the spread of α-syn from the injection site to multiple brain regions, including the medial geniculate nucleus of the thalamus, superior colliculus, periaqueductal gray matter, retrosplenial cortex and prefrontal cortex. Overexpression of α-syn was detected in all regions examined. At one-month post-injection, α-syn expression exhibited a spatial gradient, with reduced intensity observed at greater distances from the injection site (Fig. 7). Notably, α-syn levels became more pronounced by two months post-injection, with a further slight increase at the four months, indicating progressive and widespread distribution over time (Fig. 7).

### Degeneration of the nigrostriatal pathway

#### Reduction of TH + cell density in the SNpc

**Fig. 8:**
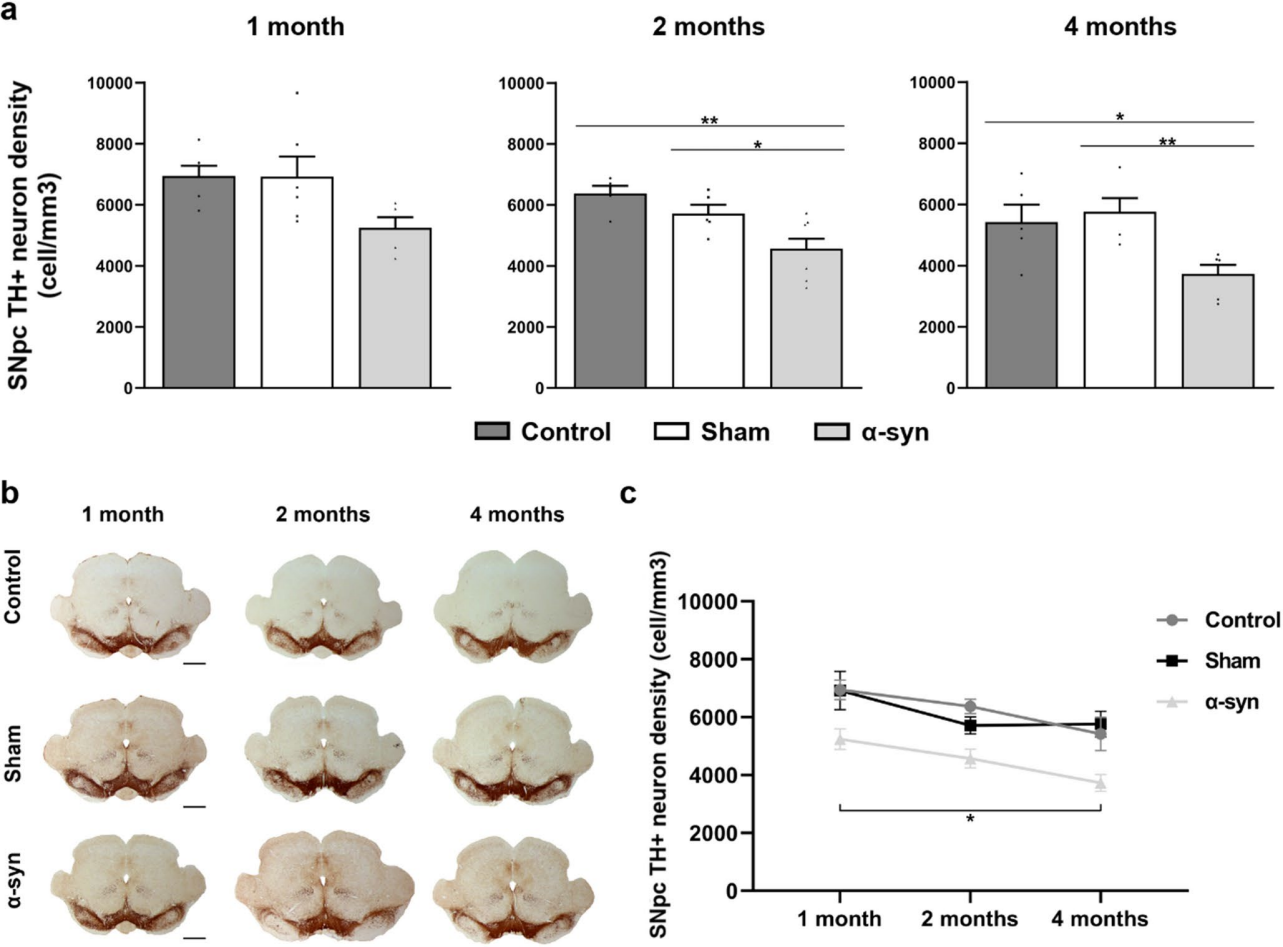
Analysis of neuronal degeneration in the SNpc across different experimental groups (control, sham, and α-syn) at all analyzed time points (one, two, and four months after injection). **(a)** Stereological quantification of TH + cell density. Data are presented as mean ± SEM. *p < 0.05 and **p < 0.01 (one-way ANOVA with post hoc corrections; *n* = 6 in each experimental group and analyzed time point). **(b)** Representative images of TH immunochemistry in the brainstem. Scale bar: 1000 μm. **(c)** Temporal analysis to assess the progressive loss of TH + cells in the SNpc. Data are presented as mean ± SEM. *(time) *p* < 0.05 (multivariate linear regression analysis)

Stereological analysis revealed a progressive reduction in DA cell density in the α-syn group compared to control and sham groups across all time points (Fig. 8a and b). One month post-surgery, the α-syn group exhibited a lower density of TH + neurons (5240 ± 354 cells/mm³) relative to the control (6942 ± 334 cells/mm³) and sham (6922 ± 658 cells/mm³) groups. By two months, this reduction became statistically significant, with the α-syn group showing a mean density of 4570 ± 325 cells/mm³, significantly lower than both the control group (6373 ± 249 cells/mm³; *p* = 0.003) and the sham group (5715 ± 293 cells/mm³; *p* = 0.049). This degenerative trend continued at four months post-injection, with the α-syn group presenting a further decline in TH + neuron density (3730 ± 292 cells/mm³), which remained significantly lower than in the control (5420 ± 576 cells/mm³; *p* = 0.031) and sham (5758 ± 443 cells/mm³; *p* = 0.008) groups (Fig. 8a and b; **Online Resource 7**). These findings support the progressive dopaminergic neurodegeneration induced by α-syn overexpression.

To assess whether temporal progression on SNpc neuronal degeneration and potential differences across experimental groups, a multivariate linear regression analysis was performed. The results demonstrated a significant effect of time on TH + cell density in the SNpc (*p* = 0.016) (Fig. 8c). In the α-syn group, neuronal density declined by 13% between one and two months post-injection, reaching a 29% by the four-month time-point. In comparison, the control group exhibited an 8% reduction at two months and a 17% decline at four months relative to one month post-injection. The sham group showed a similar pattern, with a 17% decrease observed at both two and four months (Fig. 8c). While multivariable regression did not reveal significant differences in TH + cell density progression in the α-syn group compared to control or sham groups, a trend toward more pronounced TH + cell loss was observed in the α-syn group (Fig. 8c).

#### Degeneration of TH + fibers in the striatum

**Fig. 9:**
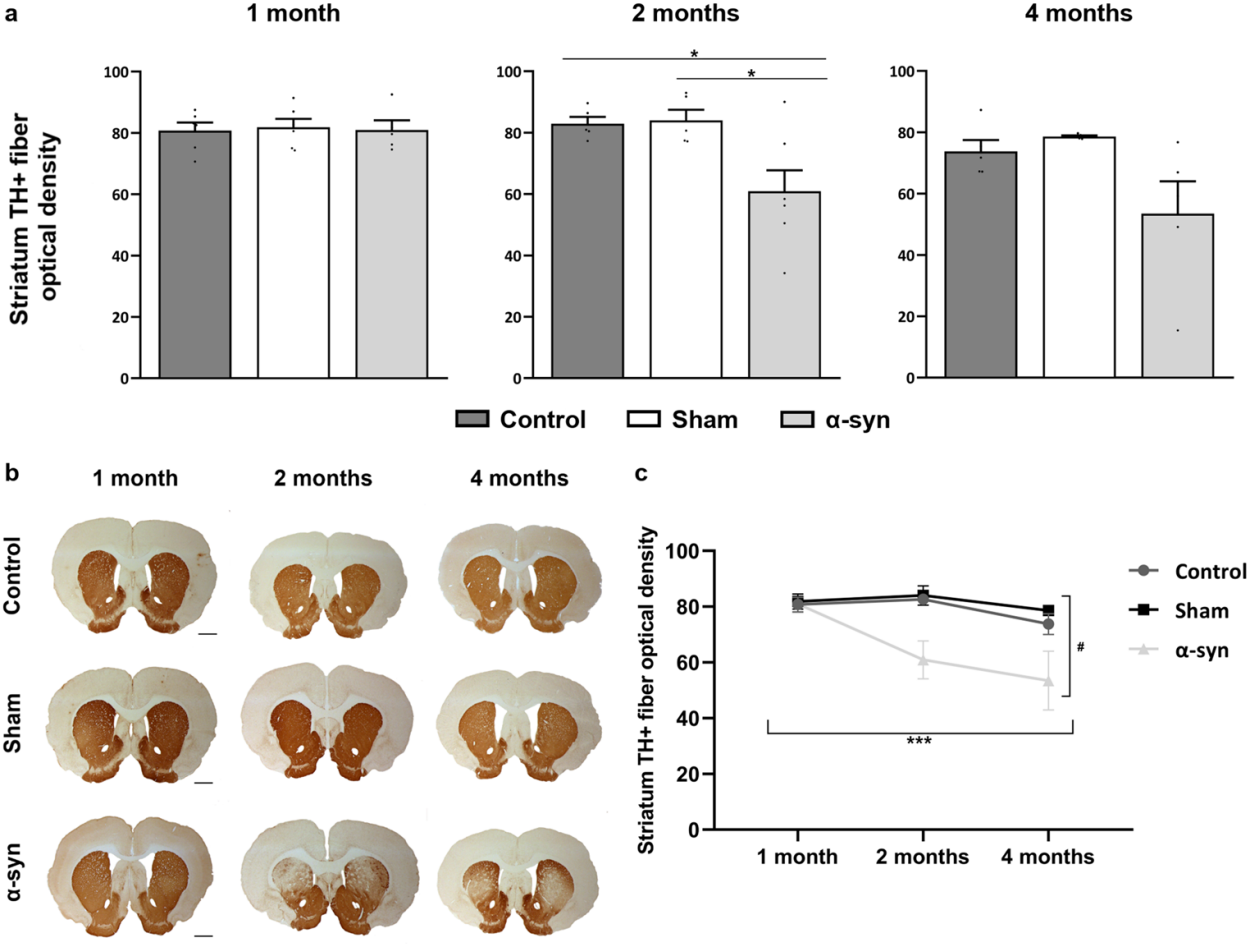
Analysis of fiber degeneration in the striatum across different experimental groups (control, sham, and α-syn) at all analyzed time points (one, two, and four months after injection). **(a)** Optical density of TH + fibres in the striatum. Data are presented as mean ± SEM. *p < 0.05 (one-way ANOVA with post hoc corrections; *n* = 6 in each experimental group and analyzed time point). **(b)** Representative images of TH immunochemistry in the striatum. Scale bar: 1000 μm. **(c)** Temporal analysis to assess the progressive loss of TH + fibres in the striatum. Data are presented as mean ± SEM. ***(time) *p* < 0.05; ^#^ (α-syn vs. sham) *p* < 0.05 (multivariate linear regression analysis)

Analysis of TH + fiber density in the striatum revealed a progressive loss in the α-syn group over time. One month post-injection, TH + fibers remained consistent across all experimental groups (control = 80 ± 2, sham = 81 ± 2, and α-syn = 80 ± 3). However, by two months, a significant reduction was observed in the α-syn group (60 ± 6) compared to the control (83 ± 2; *p* = 0.027) and sham (84 ± 3; *p* = 0.020) groups. This decline persisted at four months (53 ± 4), although no statistical significance were found compared to the control (73 ± 3, *p* = 0.341) and sham (78 ± 1, *p* = 0.211) groups (Fig. 9a and b). Multivariable regression analysis showed that time had a significant effect on striatal optical density (*p* < 0.001). In the a-syn group, TH + fiber density declined by 25% at two months and by 34% at four months post-injection compared to one month. In contrast, the sham group exhibited only a 4% reduction over the same period. Statistical analysis confirmed that the α-syn group’s fiber loss progressed significantly faster compared to the sham group (*p* = 0.046), indicating that time affects these two experimental groups differently (Fig. 9c).

#### Analysis of axonal swelling in the striatum

**Fig. 10:**
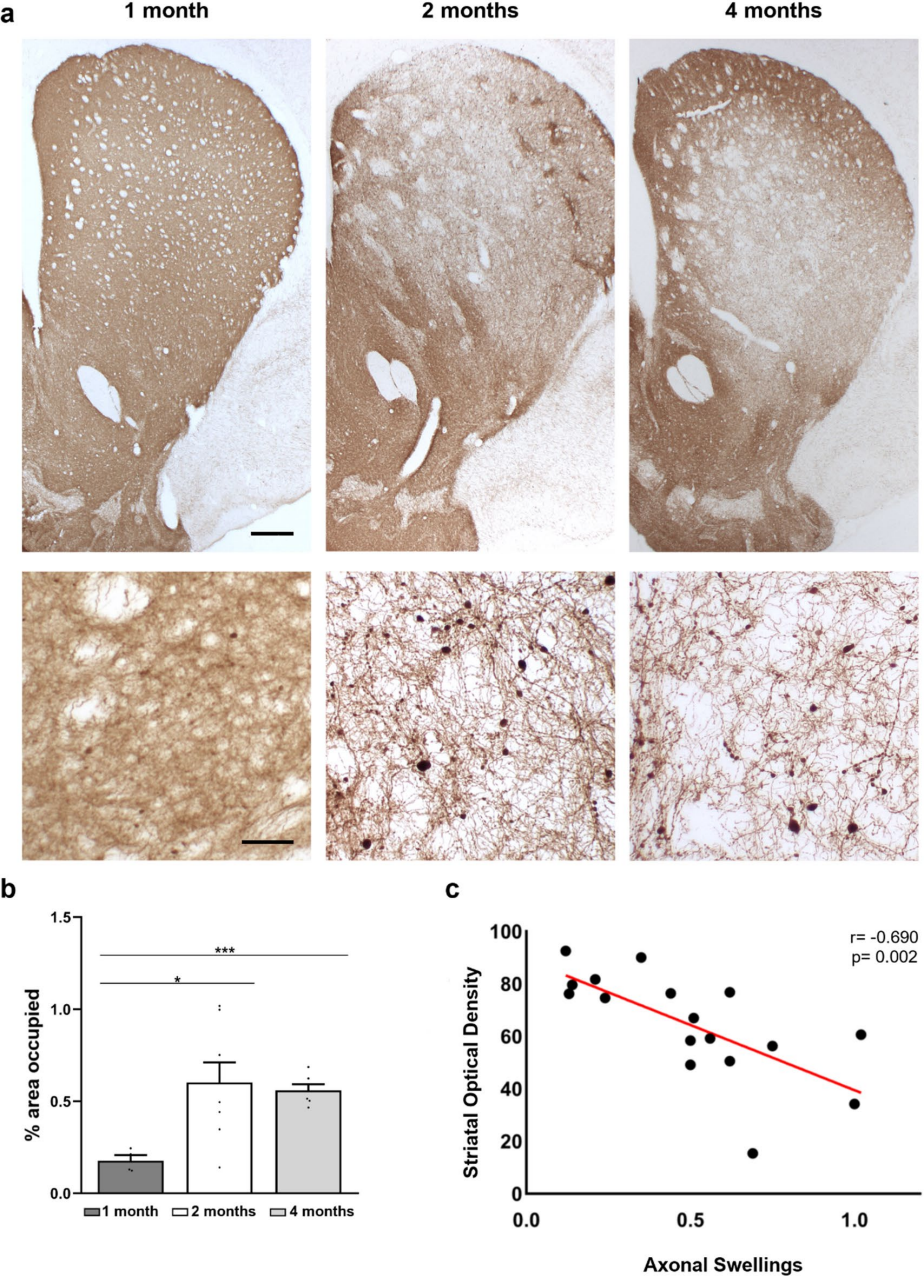
Presence of axonal swellings in the striatum. **(a)** Representative images of TH immunohistochemistry of the striatum showing both the pattern of degeneration in this nucleus and the presence of axonal swellings. Scale bar: 200 μm and 50 μm. **(b)** Quantification of the percentage of surface area occupied by axonal swellings in the striatum. Data are presented as mean ± SEM. *p < 0.05 and ***p < 0.01 (one-way ANOVA with post hoc corrections; *n* = 6 in each analyzed time point). **(c)** Correlation analysis to assess the relationship between optical density and axonal swellings in the striatum. Pearson correlation coefficient (*r*= -0.690) and p-value (*p* = 0.002) shows a negative and significant correlation (*n* = 18)

The presence of axonal swelling in the striatum of α-syn group animals was analyzed to evaluate the degenerative process. As shown in the representative images (Fig. 10a), axonal degeneration became more pronounced starting at two months post-injection and remained relatively stable though the four-month time point. Quantitative analysis supported these observations (Fig. 10b), revealing significant differences between one-month post-injection (0.17 ± 0.03%) and both the two-month (0.6 ± 0.3%; *p* = 0.017) and four-month (0.55 ± 0.08%; *p* < 0.001) time points. In addition, Pearson’s correlation coefficient demonstrated a significant inverse relationship (*r*= -0.690; *p* = 0.002) between the presence of axonal swellings and striatal optical density (Fig. 10c).

#### Reduction of DA dendrites in the SNpr

**Fig. 11:**
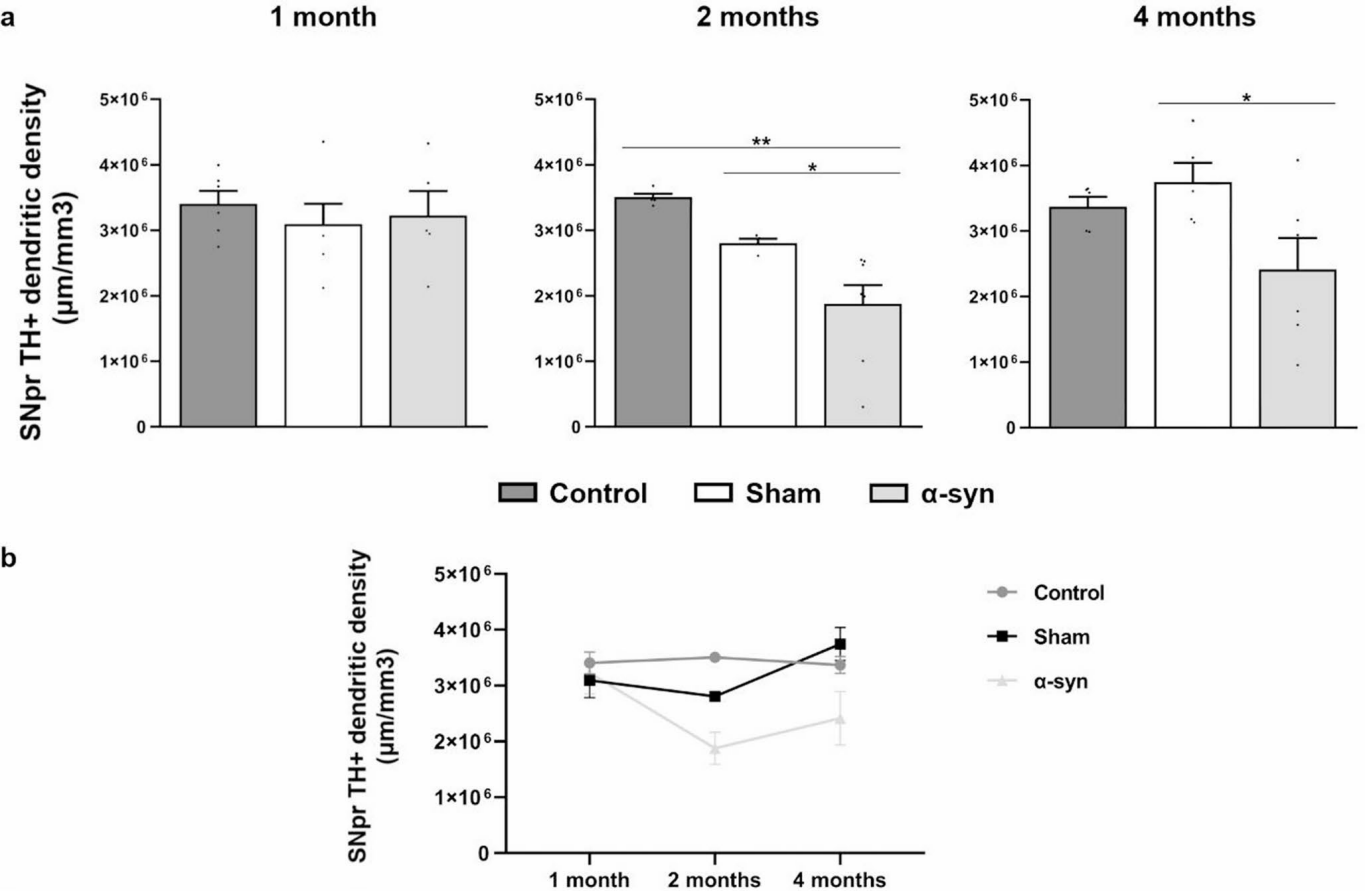
Analysis of dendritic degeneration in the SNpr across different experimental groups (control, sham, and α-syn) at all analyzed time points (one, two, and four months after injection). **(a)** Stereological quantification of TH + dendrites in the SNpr. Data are presented as mean ± SEM. *p < 0.05, **p < 0.01 (one-way ANOVA with post hoc corrections; *n* = 6 in each experimental group and analyzed time point). **(b)** Temporal analysis of TH + dendrites in the SNpr. Data are presented as mean ± SEM

The density of DA dendrites in the SNpr remained unchanged across all experimental groups at one month post-injection (control = 3.40 × 10^6^ ± 1.96 × 10^5^ μm/mm^3^; sham = 3.09 × 10^6^ ± 3.08 × 10^5^ μm/mm^3^; α-syn = 3.22 × 10^6^ ± 3.72 × 10^5^ μm/mm^3^) (Fig. 11a). However, by two months post-injection, a significant reduction was observed in the α-syn group (1.87 × 10^6^ ± 2.85 × 10^5^ μm/mm^3^) compared to both the control (3.50 × 10^6^ ± 5.15 × 10^4^ μm/mm^3^; *p* = 0.006) and sham groups (2.31 × 10^6^ ± 4.96 × 10^5^ μm/mm^3^; *p* = 0.035) (Fig. 11a). At four months post-injection, a similar trend was observed. Dendritic density in the α-syn group remained lower (2.41 × 10^7^ ± 4.78 × 10^6^ μm/mm^3^) compared to the control (3.37 × 10^7^ ± 1.53 × 10^6^ μm/mm^3^; *p* = 0.098) and sham group (3.74 × 10^7^ ± 2.95 × 10^6^ μm/mm^3^; *p* = 0.030) (Fig. 11a).

Temporal analysis revealed a 41.8% reduction in dendritic density at two months and a 25.2% reduction at four months in the α-syn group, whereas the control and sham groups exhibited only minor changes over time. Although the α-syn group appeared to exhibit greater dendritic loss than the sham group, the progression of dendritic degeneration did not differ significantly between groups (*p* = 0.076) (Fig. 11b).

### Microglial activation analysis

A qualitative and semiquantitative analysis of microglial activation was conducted to assess whether the neurodegenerative alterations observed were accompanied by changes in the inflammatory response in the SN and striatum.

**Fig. 12:**
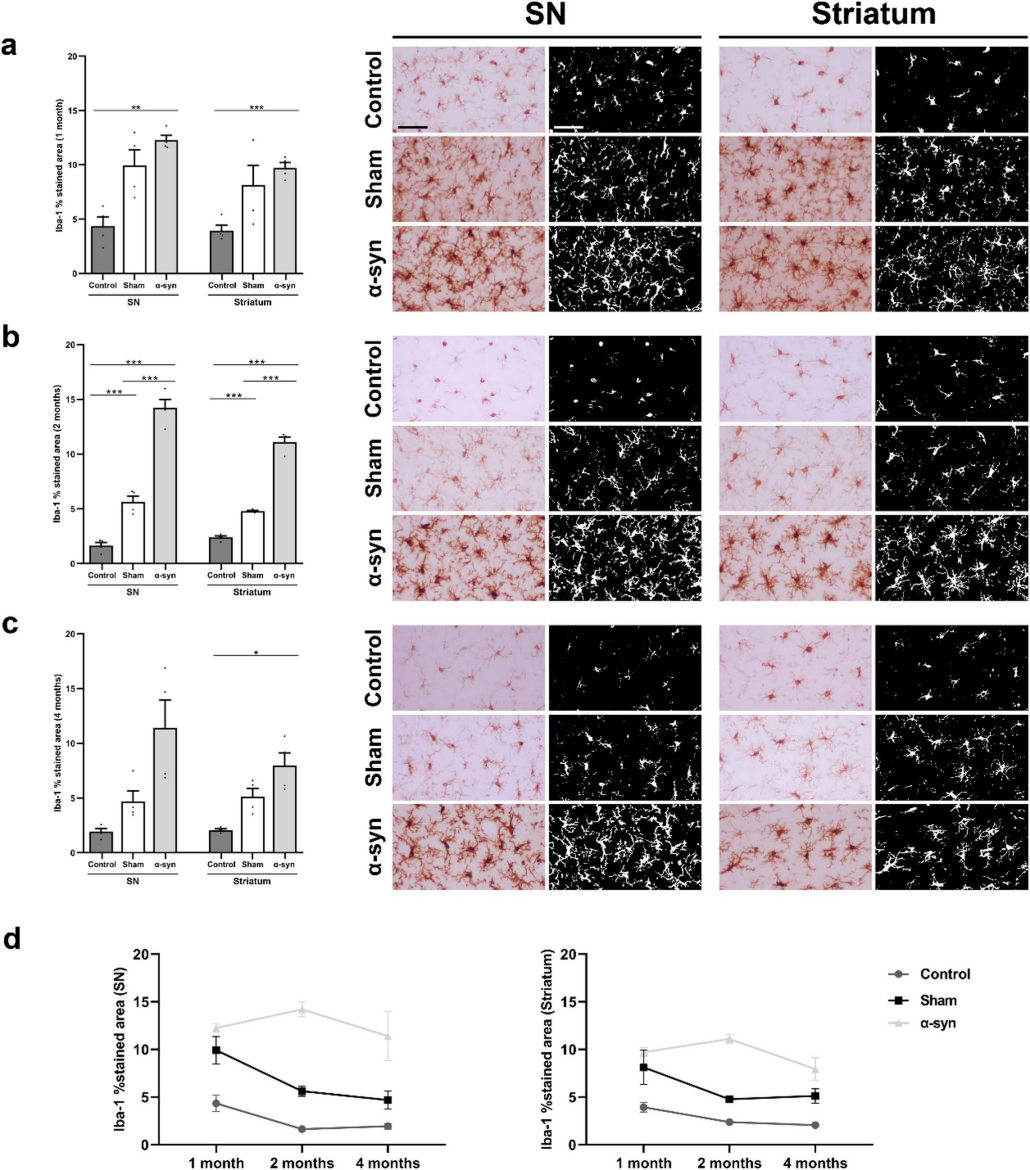
Microglial activation in the SN and striatum across different experimental groups (control, sham, and α-syn). **(a)** Quantification of the percentage of the area occupied by Iba-1 positive cells, along with representative images of Iba-1 immunohistochemistry and thresholded images at one month post-injection; **(b)** at two months post-injection; and **(c)** at four months post-injection. Data are presented as mean ± SEM. *p < 0.05, ** *p* < 0.01, ***p < 0.01 (one-way ANOVA with post hoc corrections; *n* = 4 in each experimental group and analyzed time point). Scale bar: 50 μm. **(d)** Temporal analysis assessing the progression of microglial activation. Data are presented as mean ± SEM

At one month post-injection, Iba-1 immunohistochemistry revealed a proliferation of microglia in the intervention groups, with significant differences compared to the control group. Both the sham (SN: 9.91 ± 1.44%; striatum: 8.12 ± 1.79%) and α-syn (SN: 12.25 ± 0.45%; striatum: 9.71 ± 0.49%) groups showed increased microglial activation in the SN and the striatum, compared to the control group (SN: 4.33 ± 0.86%; striatum: 3.94 ± 0.5%). Significant differences were observed between the control and α-syn groups in both regions (SN: *p* = 0.002; striatum: *p* = 0.001) (Fig. 12a). Morphological differences were also observed between the intervention groups and the control group. The α-syn and sham groups displayed hyper-ramified microglia with swollen cell bodies, while microglia in the control group retained a resting microglia (Fig. 12a).

At two months, Iba-1 positivity decreased in the sham group (SN: 5.63 ± 0.53%; striatum: 4.77 ± 0.07%). However, significant differences were still present compared to the control group (SN: 1.63 ± 0.29%; striatum: 2.38 ± 0.14%) in both the SN (*p* = 0.002) and striatum (*p* = 0.001) (Fig. 12b). In contrast, Iba-1 expression in the α-syn group remained elevated (SN: 14.21 ± 0.76%; striatum: 11.1 ± 0.45%), showing significant differences compared to both the control (SN: *p* = 0.001; striatum: *p* = 0.001) and sham groups (SN: *p* = 0.001; striatum: *p* = 0.001) (Fig. 12b). Microglial cells in the α-syn group continued to exhibit an activated morphology characterized by extensive ramification and swollen cell bodies (Fig. 12b).

At four months, no significant differences were observed between the sham (SN: 4.69 ± 0.94%; striatum: 5.12 ± 0.74%) and control (SN: 1.93 ± 0.29%, *p* = 0.157; striatum: 2.06 ± 0.12%, *p* = 0.073) groups (Fig. 12c). However, microglial activation persisted in the α-syn group (SN: 11.4 ± 2.55%; striatum: 7.94 ± 1.17%), reaching statistical significance compared to the control group in the striatum (*p* = 0.044). The morphology of Iba-1 + cells remained consistent with a similar phenotype to that described at two months post-injection **(**Fig. 12c).

Despite these baseline differences, the temporal progression of Iba-1 expression did not significantly differ between groups, as indicated by non-significant time × group interactions in both the striatum (*p* = 0.77 for control, *p* = 0.86 for sham) and SN (*p* = 0.79 for control, *p* = 0.18 for sham). Additionally, the main effect of time was not significant in either region (*p* = 0.09 for striatum; *p* = 0.43 for SN), suggesting that the observed group differences were maintained consistently over the four-month follow-up period, rather than evolving over time (Fig. 12d).

## Discussion

Different animal models have been developed to better understand the pathophysiology of PD, and the lack of appropriate models has often been considered a major obstacle in conducting preclinical evaluations of novel therapeutic interventions. Whereas most previous studies have relied on α-syn overexpression induced by unilateral AAV administration, this study employs a bilateral model. Unilateral PD models have been widely used in both neurotoxic and AAV-based approaches, and they are particularly useful for studying motor asymmetries and L-DOPA-induced dyskinesia. Unilateral models are commonly preferred in neurotoxin-based studies due to their simplicity and reduced impact on animal welfare, as bilateral lesions can lead to complications such as dysphagia and weight loss (Amalric et al. 1995). However, these models present notable limitations. One concern is that compensatory changes occur in the intact hemisphere, where increased activity and structural adaptations may partially offset motor deficits after unilateral dopamine depletion (Miklyaeva et al. 2007; Jávor-Duray et al. 2017; Kordys et al. 2017). Although these compensatory changes are especially relevant at the beginning of the disease, exploring interhemispheric communication falls beyond the scope of the present study. In contrast, bilateral models more accurately reflect the progressive nature of PD observed in patients (Zheng and Poon 2017). They produce more consistent and stable motor and non-motor phenotypes (Caudal et al. 2015; O’Donovan et al. 2020) and enable widespread and symmetrical pathology, facilitating the investigation of disease progression and neuronal interactions not accessible with unilateral approaches (Perren et al. 2016; Gubinelli et al. 2022a, b). These models are particularly valuable for assessing global network dysfunctions and for testing therapeutic strategies targeting distributed brain regions. The feasibility and reproducibility of bilateral AAV-mediated α-syn overexpression have been confirmed in multiple studies (Perren et al. 2016; Freien von Hövel et al., 2019).

The results obtained in this study demonstrate that this animal model replicates several features of PD pathophysiology, including anterograde degeneration of the nigrostriatal pathway, accompanied by axonal swelling formation and microglial activation. Neuronal degeneration following adenoviral infection begins in the soma of dopaminergic neurons of the SNpc, which are responsible for dopamine release through their axonal and dendritic projections in the striatum and SNpr, respectively, leading to the onset of the characteristic motor symptoms of the disease. This experimental model also successfully replicates the temporal progression observed in PD patients, generating a bilateral pathology that develops gradually.

### α-Synuclein overexpression occurs primarily in DA neurons and diffuses to multiple brain areas

Overexpression of α-syn was detected predominantly in dopaminergic neurons of the SNpc and striatum, with no evidence of expression in non-neuronal cell types. This was confirmed by double immunofluorescence, which showed co-localization of α-syn with TH + neurons, but not with astrocytes or microglia, supporting the neuronal specificity of the promoter used. Nevertheless, the transduction efficacy and specificity observed in the SNpc also suggest that, in addition to dopaminergic neurons, the AAV employed may transduce other neuronal populations present in the SNpc, such as GABAergic interneurons and cholinergic neurons (Nair-Roberts et al. 2008; Khudoerkov et al. 2014). Therefore, although complete specificity was not achieved, the values obtained support the viability of our animal model for overexpressing α-syn in DA neurons. In addition, despite α-syn is expressed in both DA and non-DA neurons, the degenerative changes (cell death, dystrophic axons) develop only in the DA neurons (Van der Perren et al. 2015; Björklund and Mattsson 2024).

Additionally, the co-localization persisted across all analyzed time points, suggesting the suitability of AAVs for inducing α-syn overexpression in DA neurons. These findings are consistent with previous studies reporting α-syn and TH co-expression from three weeks (Koprich et al. 2010) up to three months (Decressac et al. 2012; Gombash et al. 2013; Ip et al. 2017). Furthermore, the progressive reduction in TH/α-syn co-expressing neurons and fibers over time suggests that α-syn overexpression contributes to dopaminergic cell loss and axonal degeneration, findings that support the ongoing nigrostriatal pathway degeneration observed in this model.

Immunofluorescence assays further demonstrated that α-syn overexpression extends beyond the nigrostriatal pathway, spreading to multiple brain regions over time. The spatial distribution of α-syn exhibited a spatial gradient, with expression levels diminishing in proportion to the distance from the injection site increases. Notably, α-syn accumulation became more pronounced at two months post-injection, indicating a progressive propagation within the brain. The observed spatiotemporal distribution pattern in our model aligns with the progression of α-syn pathology described in the human central nervous system by Braak et al. (2004). Although cortical α-syn overexpression was not initially present, evidence from in vitro studies suggest that neurons are capable of secreting and internalizing α-syn from the extracellular space, supporting a prion-like, cell-to-cell transmission mechanism (Gómez-Benito et al. 2020). The spread of α-syn to additional brain areas may disrupt their functionality, highlighting the need for future studies to evaluate the functional consequences of α-syn accumulation beyond the nigrostriatal system.

### Impaired motor activity induced by α-syn overexpression

Progressive motor impairments were induced by α-syn overexpression, as evidenced by significant reductions in overall activity, locomotor activity, and mean velocity over time. These behavioral alterations became evident one month post-injection and persisted throughout the observation period, peaking at two months post-injection. Previous studies have reported a significant reduction in distance traveled (Campos et al. 2013; Oliveras-Salvá et al. 2013), an increase in resting time (Campos et al. 2013), and a marked decrease in locomotor activity (Ekmark-Lewen et al., 2018) in animals overexpressing α-syn. The observed motor impairments could be attributed to α-syn-induced degeneration of DA neurons in this model, affecting the dopamine release in the striatum and in SNpr, thereby causing the described motor alterations (Nikolaus et al. 2003; Day et al. 2006).

Although these impairments reached statistical significance at one and two months, they were no longer distinguishable between experimental groups at four months, likely due to a general decline in activity across all groups. The absence of differences at this time point may be related to the significant negative correlation observed between weight gain and motor behavior, suggesting that changes in weight could contribute to the decline in motor performance. Indeed, during this period, an increase in body weight was recorded, and other studies have also reported an association between weight gain and reduced activity in animals (Spangenberg et al. 2005; Maric et al. 2022).

### α-Synuclein overexpression led to a progressive dopaminergic degeneration

This study demonstrates that α-syn overexpression leads to a progressive loss of dopaminergic neurons in the SNpc. Significant neuronal degeneration is evident by two months post-injection and worsens at four months, indicating an ongoing neurodegenerative process. These findings are consistent with previous reports showing a reduction of TH + cells in the SNpc after AAV-mediated α-syn delivery in rats (Decressac et al. 2012; Gombash et al. 2013; Negrini et al. 2022). Similar dopaminergic loss has also been observed in non-human primates, 12 weeks after adenoviral injection into the SNpc of *Macaca fascicularis* (Sucunza et al. 2021). Although some degree of neuronal loss occurred across all groups over time, the α-syn group showed a significantly greater decline, suggesting that α-syn overexpression accelerates dopaminergic neurodegeneration.

Concomitantly, a marked reduction in striatal fiber density was observed in the α-syn group, particularly at two months post-injection, while no significant changes were detected at one month. This temporal pattern suggests that degeneration begins in the SN and progressively affects the striatum via the nigrostriatal pathway. Similar dynamics have been reported in 6-OHDA models, where neuronal loss in the SN precedes striatal fiber degeneration (Haleagrahara et al. 2013). Although fiber loss persisted at four months post-injection, statistical significance was not reached, indicating a plateau in α-syn-induced degeneration.

In AAV-based models, the level of transgene expression can determine neuronal fate, with some neurons persisting in a dysfunctional state rather than undergoing immediate degeneration (Negrini et al. 2022). In such cases, α-syn pathology is initially characterized by axonal and terminal alterations, including a population of TH + fibers with dystrophic swellings. These fibers retain TH immunoreactivity but exhibit impaired function due to the accumulation of phosphorylated α-syn aggregates (pSer129), indicative of presynaptic dysfunction (Negrini et al. 2022).

Consistent with this, our study identified axonal swellings in the striatum of α-syn-expressing animals, which became more prominent from two months onward and progressively increased over time. A strong negative correlation between axonal swellings and TH + optical density further supports their association with striatal tissue degeneration. These findings align with previous studies reporting swollen neurites (Van der Perren et al. 2015), reductions in α-syn-positive fibers, and time-dependent increases in axonal dilations (Phan et al. 2017; Gubinelli et al. 2022a). Morphologically, these swellings resemble axonal spheroids seen in Parkinson’s disease patients, which result from impaired axonal transport and represent a transitional stage prior to axonal fragmentation (Zhou et al. 1998; Tagliaferro and Burke 2016). Thus, the increase in TH + swellings observed here supports the notion that optical density measurements may underestimate the extent of striatal dysfunction. Despite this, our results clearly show concurrent reductions in TH + optical density and increases in axonal swellings, indicating ongoing striatal pathology in α-syn-overexpressing animals. Moreover, these findings are consistent with previous studies showing that motor symptoms in AAV-based models result not only from the loss of dopaminergic neurons but also from the dysfunction of surviving, yet dystrophic, neurons (Björklund and Mattsson 2024).

Furthermore, the neurotoxic effect of α-syn overexpression in nigral dopaminergic neurons is dose-dependent (Björklund and Mattsson 2024). While rat models have employed AAV-α-syn vectors at working titers ranging from 10¹² to 10¹⁴ gc/ml, transduction efficiency can be affected by multiple factors including vector serotype, injection parameters, promoter strength, and enhancer elements (Björklund and Mattsson 2024). Consequently, the severity of degeneration likely depends on the effective transgene expression achieved in each model. Indeed, studies have reported nigrostriatal degeneration twice as severe as that observed here when using double the viral volume (Koprich et al. 2010; Decressac et al. 2012; Gombash et al. 2013). Conversely, other studies employing larger volumes than ours have reported similar levels of neuronal loss (Ip et al. 2017; Negrini et al. 2022). These findings reinforce the view that α-syn overexpression induces nigrostriatal degeneration in a dose dependent-manner and underscore the importance of calibrating expression levels in each animal model.

Stereological analysis also revealed a significant reduction in dopaminergic dendritic density in the SNpr beginning at two months post-injection, persisting through four months. These findings are consistent with previous studies that report similar decreases at 3 weeks, with a progressive decline between 8 and 16 weeks post-injection (Li et al. 2009; Ramonet et al. 2011; Decressac et al. 2012), as well as in transgenic mice (Tagliaferro et al. 2015). Recently, it has been reported that while striatal dopamine depletion alone induces motor learning deficits and fine motor impairment, it is insufficient to produce the gross motor deficits characteristic of clinical parkinsonism (Gonzalez-Rodriguez et al., 2021). According to this study, somatodendritic dopamine loss, in addition to striatal depletion, is crucial for the development of Parkinson’s-like gait deficits in mice. Similarly, Lopez-Gonzalez del Rey et al. (2024) observed specific motor symptoms in monkeys following nearly complete loss of ventral neurons in the SNpc and their dendritic projections to the SNpr. These findings further support our results, where reduced dopaminergic density in the SNpr and striatum correlates with worsening motor function in our animal model.

Taken together, these findings indicate that α-syn overexpression exerts a progressive neuronal degeneration and loss of dopaminergic fibers in the striatum and dendrites in the SNpr, a hallmark of PD.

### Increased microglial reactivity in the nigrostriatal pathway after α-syn overexpression

In our experimental PD model, results revealed an increase in the area occupied by Iba-1-positive staining in the SN of operated animals (sham and α-syn) one month post-injection. This microglial proliferation was characterized by significant morphological changes, including increased branching and swollen cell bodies, consistent with an activated microglia phenotype. In contrast, the control group displayed morphological features consistent with resting microglia. These diferrences between operated and non-operated groups likely reflect inflammatory resonse to intracranial AAV injection. Indeed, hyper-ramified microglial phenotypes and increased process length have been reported in animal models of traumatic brain injury and post-traumatic stress one month after the injury (Smith et al. 2019; Grovola et al. 2023). Moreover, it is consistent with our finding that, by two months post-injection, the Iba-1-positive area in the sham group was comparable to that of the control group.

Previous studies in PD patients have reported increased nigral microglia (Gerhard et al. 2006; Smajić et al. 2022), supporting the notion that microglia play a central role in PD-associated neuroinflammation (Moehle and West 2015). In our study, α-syn overexpression induced sustained microglial proliferation along the nigrostriatal pathway, persisting up to four months post-injection, with the most pronounced activation observed in the SN. Similar findings have been described in other animal models of PD, where an increase in the glial population has been described in both neurotoxic (Farrand et al. 2017) and transgenic models (Su et al. 2008; Watson et al. 2012).

Moreover, Iba-1-positive cells in the α-syn group consistently exhibited hyper-ramification and enlarged cell bodies, features indicative of activated microglia. It has been demonstrated that α-syn aggregates can activate glial cells, and that α-syn-induced neuronal pathology plays a crucial role in modulating microglial phenotype (Sanchez-Guajardo et al. 2010; Barkholt et al. 2012). Similar morphological transformations, marked by hypertrophic and highly branched microglia, have also been observed in models of neuroinflammation in the cerebral cortex (Kwon et al. 2018).

These findings underscore the progression of microglial activation and morphological remodeling in the presence of α-syn pathology, along with the persistent differences among the experimental groups.

### Limitations

This study presents certain limitations. The exclusive use of male rats limits generalizability due to known sex-related differences in neurodegeneration and therapeutic responses. The selected time points allowed the characterization of early and progressive changes, but longer observation periods may be required to capture more advanced stages of the pathology. Nonetheless, the use of shorter timeframes offers a practical advantage for accelerating the evaluation of potential therapies. Methodologically, one limitation of this model is that the AAV vector transduces not only DA neurons but also non-DA neurons in the SNpc. However, degenerative changes occur in DA neurons. The assessment of striatal optical density may also capture dysfunctional fibers in the α-syn group; thus, complementary biochemical assays would provide a more accurate measure of functional fibers. Finally, while α-syn overexpression replicates key pathological hallmarks of PD, it does not fully recapitulate the complex patterns of disease propagation seen in humans. Despite these limitations, the model successfully induces progressive neurodegeneration and represents a valuable tool for elucidating disease mechanisms and evaluating therapeutic strategies in a controlled and time-efficient manner.

## Conclusions

Our results demonstrate that α-syn overexpression, induced by bilateral administration of rAAV9-CMVie/SynP-WPRE, triggers selective dopaminergic degeneration, initially affecting the somas of DA neurons in the SNpc and progressively extending to projection nuclei and other brain regions. Both the spread of α-syn and the associated neurodegeneration follow a temporally progressive pattern. Notably, morphological analyses reveal significant impairments from two months post-injection onward, coinciding with a reduction in dopaminergic neurons and fibers, an increased presence of axonal swellings, enhanced glial activation, and a deterioration in motor function.

Temporal analysis identifies the two-month post-injection time point as the optimal window for intervention and for evaluating potential neuroprotective strategies targeting α-syn-induced pathology, but should be independently validated within each study.

## Electronic supplementary material

Below is the link to the electronic supplementary material.

Supplementary Material 1

Supplementary Material 2

Supplementary Material 3

Supplementary Material 4

Supplementary Material 5

Supplementary Material 6

## Data Availability

No datasets were generated or analysed during the current study.
