# Supplementary material for: Temporal progression of pathological features in an α-synuclein overexpression model of Parkinson’s disease
Source: Brain Struct Funct. 2025 Jun 9;230(6):91. doi: 10.1007/s00429-025-02959-9 (PMC12149260; doi:10.1007/s00429-025-02959-9)
Supplement: Supplementary file 1 — Supplementary Material 1 [file 429_2025_2959_MOESM1_ESM.pdf]

Article title: **Temporal Progression of Pathological Features in an α-Synuclein Overexpression Model of Parkinson's Disease**

Journal: Brain Structure and Function

Authors names: Andrea Vaquero-Rodríguez, Jone Razquin, Ane Murueta-Goyena, Cristina Miguelez, José Ángel Ruíz-Ortega, José Vicente Lafuente, Harkaitz Bengoetxea and Naiara Ortuzar

Corresponding author: Harkaitz Bengoetxea (harkaitz.bengoetxea@ehu.eus).

Department of Neurosciences, Faculty of Medicine and Nursing, University of the Basque Country (UPV/EHU), 48940 Leioa, Spain

Neurodegenerative Diseases Group, Biobizkaia Health Research Institute, 48903 Barakaldo, Spain

ONLINE RESOURCE 1 & 2

Online Resource 1 Table shows the results of the Open Field test**,** which was used to evaluate motor impairments in the control, sham, and α-syn groups at one, two, and four months post-injection. Data are presented as the mean ± SEM.

		**Control**	**Sham**	**α-syn**
**Global activity**	1 month	2120 ± 136	2402 ± 149	1905 ± 126
	2 months	2123 ± 133	2065 ± 143	1570 ± 130
	4 months	1654 ± 96	1445 ± 69	1372 ± 98
**Locomotion**	1 month	1895 ± 123	2167 ± 133	1718 ± 111
	2 months	1900 ± 121	1828 ± 125	1381 ± 112
	4 months	1469 ± 89	1272 ± 61	1213 ± 76
**Mean velocity (cm/s)**	1 month	9.24 ± 0.63	9.67 ± 0.87	7.49 ± 0.67
	2 months	9.26 ± 0.63	8.82 ± 0.73	6 ± 0.70
	4 months	7.53 ± 0.44	6.42 ± 0.39	5.85 ± 0.62

Online Resource 2 Table presents the significant differences between the experimental groups (*p* values) using a one-way analysis of variance (ANOVA) with Tukey's post-hoc corrections for homogeneous variances, or Tamhane T2 for heterogeneous variances (SPSS Statistics 29.0, IBM, Spain).

		**α-syn vs. control**	**α-syn vs. sham**	**sham vs. control**
**Global activity**	1 month	> 0.05	**0.029**	> 0.05
	2 months	**0.020**	**0.018**	> 0.05
	4 months	> 0.05	> 0.05	> 0.05
**Locomotion**	1 month	> 0.05	**0.007**	> 0.05
	2 months	**0.009**	**0.016**	> 0.05
	4 months	> 0.05	> 0.05	> 0.05
**Mean velocity (cm/s)**	1 month	> 0.05	> 0.05	> 0.05
	2 months	**0.012**	**0.016**	> 0.05
	4 months	> 0.05	> 0.05	> 0.05
