# Supplementary material for: Temporal progression of pathological features in an α-synuclein overexpression model of Parkinson’s disease
Source: Brain Struct Funct. 2025 Jun 9;230(6):91. doi: 10.1007/s00429-025-02959-9 (PMC12149260; doi:10.1007/s00429-025-02959-9)
Supplement: Supplementary file 3 — Supplementary Material 3 [file 429_2025_2959_MOESM3_ESM.pdf]

Article title: Temporal Progression of Pathological Features in an α-Synuclein Overexpression Model of Parkinson's Disease

Journal: Brain Structure and Function

Authors names: Andrea Vaquero-Rodríguez, Jone Razquin, Ane Murueta-Goyena, Cristina Miguelez, José Ángel Ruíz-Ortega, José Vicente Lafuente, Harkaitz Bengoetxea and Naiara Ortuzar
Corresponding author: Harkaitz Bengoetxea (harkaitz.bengoetxea@ehu.eus). Department of Neurosciences, Faculty of Medicine and Nursing, University of the Basque Country (UPV/EHU), 48940 Leioa, Spain.
Neurodegenerative Diseases Group, Biobizkaia Health Research Institute, 48903 Barakaldo, Spain

Online Resource 7 Table shows stereological estimates for each individual animal of all experimental groups and analyzed time points. N represent the estimated number of TH+ cells in substantia nigra in each animal. The sampling parameter were the same for all animals, including a distance between sections of 480 µm, 50x50 μm^2 counting frame area, 120x120 μm^2 sampling grid area and 95% of dissector height.

Group	Animal	no. of sections	no. of sampling sites	no. cells counted	N	CE	Volume (mm^3)	Cell density (no./mm^3)
1-month								
asyn	1	8	739	590	22899	0.09	5	4.237
asyn	2	8	682	742	29727	0.09	5	6.055
asyn	3	9	832	576	26727	0.06	6	4.600
asyn	4	8	821	651	31330	0.09	6	5.448
asyn	5	8	717	655	29562	0.09	5	5.865
sham	1	8	579	895	32206	0.12	4	7.972
sham	2	8	308	523	20484	0.09	2	9.660
sham	3	8	390	439	17172	0.12	3	6.256
sham	4	8	551	544	22743	0.06	4	5.628
sham	5	7	423	370	15350	0.06	3	5.458
sham	6	7	466	612	22488	0.11	3	6.564
control	1	8	649	852	33174	0.08	5	7.370
control	2	8	573	660	25678	0.09	4	6.280
control	3	8	564	728	28065	0.08	4	6.926
control	4	7	620	923	35808	0.09	4	8.132
control	5	8	442	454	17524	0.09	3	5.807
control	6	7	667	832	33140	0.11	5	7.137

Group	Animal	no. of sections	no. of sampling sites	no. cells counted	N	CE	Volume (mm^3)	Cell density (no./mm^3)
2-months								
asyn	6	7	403	398	14690	0.06	3	5.730
asyn	7	8	475	403	17495	0.09	3	5.427
asyn	8	8	613	412	20924	0.07	4	4.738
asyn	9	8	570	704	21119	0.09	4	5.353
asyn	10	7	627	618	15644	0.07	4	3.513
asyn	11	8	722	465	20278	0.06	5	3.923
asyn	12	8	728	442	17220	0.08	5	3.280
sham	7	8	452	409	16047	0.09	3	5.445
sham	8	8	549	498	19719	0.06	4	4.879
sham	9	8	432	589	20889	0.06	3	6.252
sham	10	7	528	536	21898	0.08	4	5.503
sham	11	8	521	655	25755	0.09	4	6.498
control	7	8	655	780	32221	0.08	5	6.875
control	8	7	476	631	22698	0.07	3	6.538
control	9	7	503	514	19744	0.09	4	5.449
control	10	8	631	691	30057	0.07	4	6.704
control	11	7	622	669	27168	0.06	4	6.300

Group	Animal	no. of sections	no. of sampling sites	no. cells counted	N	CE	Volume (mm^3)	Cell density (no./mm^3)
4-months								
asyn	25	6	623	415	19460	0.08	4	4.364
asyn	26	8	651	417	19046	0.07	5	4.200
asyn	27	8	584	413	17309	0.05	4	4.178
asyn	28	7	523	368	10401	0.08	4	2.741
asyn	29	8	631	412	17848	0.07	4	4.004
asyn	30	7	535	433	11162	0.1	4	2.895
sham	25	8	669	856	34652	0.07	5	7.217
sham	26	8	661	688	27341	0.08	5	5.758
sham	27	8	652	677	27800	0.07	5	6.109
sham	28	7	678	458	23029	0.06	5	4.691
sham	29	8	718	637	25349	0.06	5	5.017
control	25	7	859	586	31220	0.1	6	4.893
control	26	7	655	657	24077	0.1	5	5.201
control	27	8	672	849	34137	0.1	5	7.011
control	28	8	801	703	34729	0.07	6	6.309
control	29	7	570	383	14781	0.12	4	3.688
