# Supplementary material for: Temporal progression of pathological features in an α-synuclein overexpression model of Parkinson’s disease
Source: Brain Struct Funct. 2025 Jun 9;230(6):91. doi: 10.1007/s00429-025-02959-9 (PMC12149260; doi:10.1007/s00429-025-02959-9)
Supplement: Supplementary file 4 — Supplementary Material 4 [file 429_2025_2959_MOESM4_ESM.pdf]

Article title: Temporal Progression of Pathological Features in an α-Synuclein Overexpression Model of Parkinson's Disease

Journal: Brain Structure and Function

Authors names: Andrea Vaquero-Rodríguez, Jone Razquin, Ane Murueta-Goyena, Cristina Miguelez, José Ángel Ruíz-Ortega, José Vicente Lafuente, Harkaitz Bengoetxea and Naiara Ortuzar

Corresponding author: Harkaitz Bengoetxea (harkaitz.bengoetxea@ehu.eus).

Department of Neurosciences, Faculty of Medicine and Nursing, University of the Basque Country (UPV/EHU), 48940 Leioa, Spain

Neurodegenerative Diseases Group, Biobizkaia Health Research Institute, 48903 Barakaldo, Spain

ONLINE RESOURCE 4

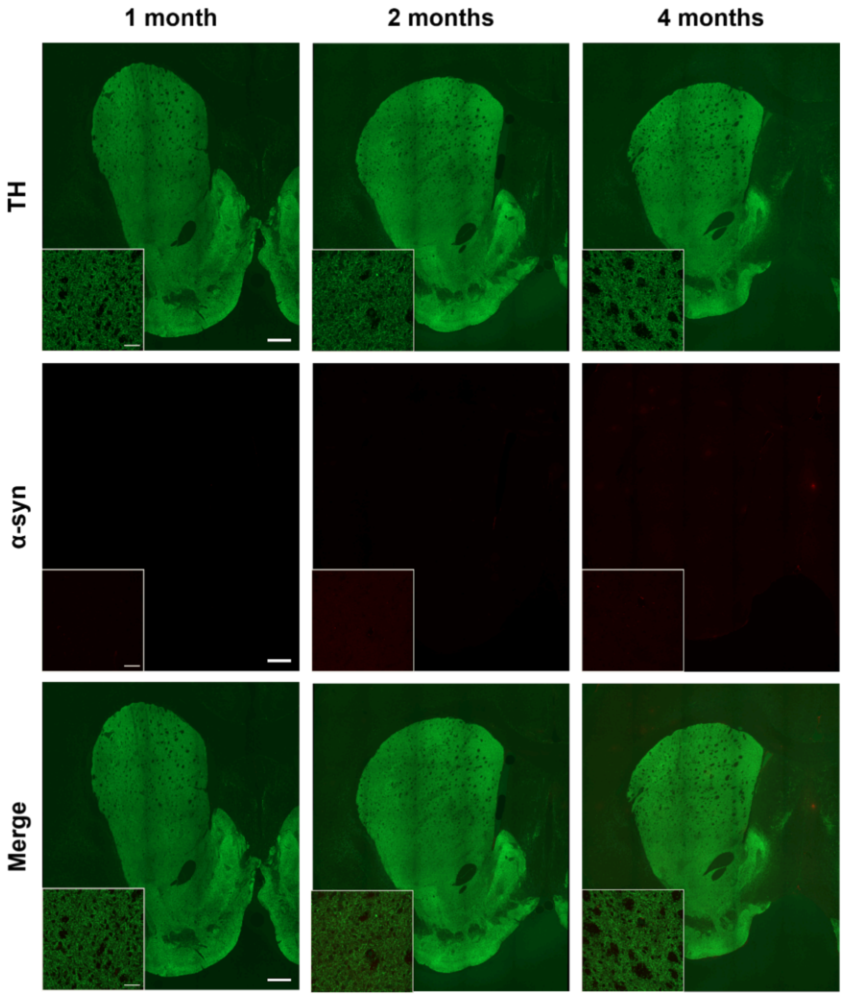

Online Resource 4 Representative double immunofluorescence images of the striatum from control animals showing positive labelling for TH (green), α-syn (red), and their co-expression (yellow) at one, two, and four-months post-injection (n=2 in each analyzed time point) Scale bar: 500 μm and 50 μm
